# ProRB: a structure-free unified framework for joint prediction and design of protein–RNA interactions

**DOI:** 10.1093/nar/gkag870

**Published:** 2026-09-07

**Authors:** Yiming Xue, Xiaojian Liu, Weimin Zhu, Shengfan Wang, Hong-Bin Shen, Xiaoyong Pan

**Affiliations:** Institute of Image Processing and Pattern Recognition, Shanghai Jiao Tong University, and Key Laboratory of System Control and Information Processing, Ministry of Education of China, Shanghai 200240, China; Institute of Image Processing and Pattern Recognition, Shanghai Jiao Tong University, and Key Laboratory of System Control and Information Processing, Ministry of Education of China, Shanghai 200240, China; Institute of Image Processing and Pattern Recognition, Shanghai Jiao Tong University, and Key Laboratory of System Control and Information Processing, Ministry of Education of China, Shanghai 200240, China; Institute of Image Processing and Pattern Recognition, Shanghai Jiao Tong University, and Key Laboratory of System Control and Information Processing, Ministry of Education of China, Shanghai 200240, China; Institute of Image Processing and Pattern Recognition, Shanghai Jiao Tong University, and Key Laboratory of System Control and Information Processing, Ministry of Education of China, Shanghai 200240, China; Institute of Image Processing and Pattern Recognition, Shanghai Jiao Tong University, and Key Laboratory of System Control and Information Processing, Ministry of Education of China, Shanghai 200240, China

## Abstract

While protein–RNA interactions are fundamental to post-transcriptional processes, achieving a holistic understanding of their regulatory logic remains challenging. Current computational models often treat binding affinity, interface mapping, and RNA design as isolated tasks, thereby failing to provide a unified perspective of the protein–RNA interactome. Here, we introduce ProRB, a unified sequence-based framework that jointly estimates protein–RNA binding affinity, predicts binding interfaces in proteins and RNAs, and generates protein-binding RNA sequences from protein sequences. By fusing protein and RNA embeddings from language models via adaptive cross-modal attention, ProRB learns contextual and relational features for predicting protein–RNA binding affinity and interface contacts, outperforming or achieving competitive performance compared to structure-based methods. Notably, its cross-attention maps reveal interpretable, motif-centric binding logic hidden in protein–RNA interactions. Building on this interpretability, ProRB enables computationally prioritized design of protein-binding RNA sequences with enhanced biophysical properties and functional motifs. By unifying the prediction, interpretation, and generation tasks, ProRB provides a scalable unified model for decoding the protein–RNA interaction and engineering motif-guided RNA therapeutics.

## Graphical Abstract

**Graphical Abstract:**
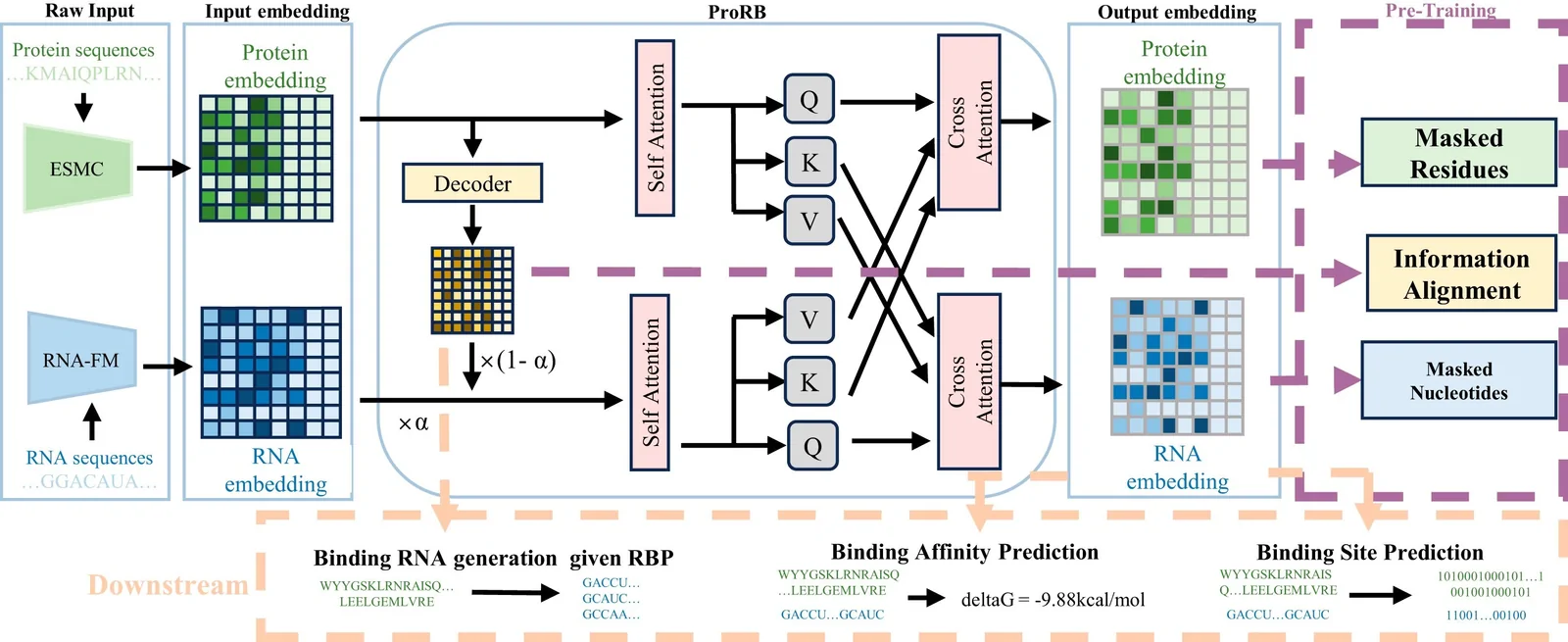

## Introduction

Protein–RNA interactions are pivotal for post-transcriptional gene regulation, orchestrating processes [1]. RNA-binding proteins (RBPs) form dynamic complexes with target RNAs, where the binding affinity and site specificity dictate regulatory outcomes [2, 3]. Dysregulation of these interactions underlies numerous human diseases, including cancer, neurodegeneration, and viral infections [1]. For instance, aberrant RBP activity contributes to over 20% of genetic disorders linked to RNA metabolism [4].

High-throughput techniques like cross-linking immunoprecipitation sequencing (CLIP-seq) and RNAcompete have illuminated RBP motifs and binding landscapes. Separate computational models can be developed to infer protein–RNA binding affinity and interfaces, facilitating protein-binding RNA sequence design. Protein–RNA binding affinity prediction [5–9] focuses on estimating the thermodynamic strength (e.g. *Δ G*) of interactions from either sequences or structures, guiding the prioritization of potential regulatory targets. Protein–RNA binding interface prediction [10–12] involves identifying binding sites at both the amino acid and nucleotide levels, revealing the positional determinants of binding specificity for targeted mutagenesis studies [13]. For protein-binding RNA sequence design, it seeks to generate *de novo* RNA sequences that bind a given RBP with desired stability and motif fidelity [14–16], supporting applications in synthetic biology and therapeutics. Moreover, the three tasks are interconnected: binding interfaces shape structural constraints that influence the affinity, while short sequence motifs guide specific recognition and binding strength. Integrating them into a unified framework reveals how conserved motifs align interfaces and tune affinity, supporting reliable, interpretable RNA therapeutic design.

Current computational protein–RNA prediction methods are broadly divided into structure-based and sequence-based categories. Structure-based models, such as CoPRA [5] and FAFormer [17], leverage 3D atomic coordinates from PDB structures to infer the binding affinities and interfaces via geometric heuristics or graph neural networks. These structure-based methods may offer physical interpretability, capturing nuanced spatial dependencies, such as the geometric constraints of atomic arrangements and inter-residue distances. However, they are constrained by the scarcity of high-resolution complex structures—only ∼3 600 high-resolution protein–RNA complexes are available in the PDB, spanning the limited diversity in RBPs and RNA motifs. This data sparsity leads to the poor generalization, particularly for *de novo* design of RBPs with limited structural templates, such as LIN28A, where high-resolution complex structures are scarce. Moreover, these models often assume rigid interfaces, overlooking RNA conformational flexibility and induced-fit mechanisms [18].

In contrast, earlier sequence-based predictors primarily relied on hand-crafted biophysical features and classical machine learning algorithms. For instance, PRA-pred [19] utilizes structural and physicochemical properties of residues, while PRdeltaGPred [20] incorporates pseudo-amino acid composition to capture evolutionary information. Similarly, PredPRBA [21] employs a web-based ensemble approach to estimate binding affinities. These methods often depend on pre-defined feature engineering, which may fail to capture the complex, non-linear dependencies inherent in cross-modal protein–RNA interactions. Recently, deep learning-based methods like DeePNAP [6], which extracts biophysical features or simple one-hot sequence encoding and leverages Transformer or other deep architectures to capture sequential information for protein–RNA binding affinity prediction, exploit evolutionary patterns with richer data. Large-scale multi-omic sequence transformers, such as OmniBioTE [22], demonstrate the potential of modeling protein–nucleic acid interactions via unified sequence pretraining. For *de novo* binding RNA generation given protein sequences, several sequence-based generative models have been proposed, such as RNAGEN [15], GenerRNA [14], and RNAtranslator [16]. Despite their generative capabilities, these methods primarily rely on high-throughput but noisy experimental data (e.g. CLIP-seq and RNAcompete [23]). While these sources provide a wealth of sequence information, they often suffer from low signal-to-noise ratios and lack the atomic-level labels derived from structural complexes. Consequently, models trained on these data tend to prioritize capturing statistical sequence distributions rather than internalizing the rigorous biophysical constraints of native interactions.

Crucially, current methods model these three tasks separately, precluding a unified understanding of the protein–RNA interactions, as the selection of binding sites and the comprehension of key binding motifs fundamentally underpin accurate binding affinity predictions. Indeed, the binding affinity and sites directly influence the biophysical plausibility, functional rationality, and biological robustness of designed sequences. Beyond task isolation, current approaches for predicting binding sites are mainly limited to single-modality analyses (e.g. protein-only [10, 11] or RNA-only [12] interfaces), which fail to capture knowledge specific to protein–RNA complexes. While recent state-of-the-art all-atom structure predictors, such as AlphaFold 3 (AF3) [24] and Boltz-1 [25], have significantly advanced modeling 3D molecular interactions, they often suffer from high computational overhead. Although models like FAFormer [17] have explored contact map prediction using much less computational cost, a gap remains in developing unified sequence-only architectures that can internalize structure-verified interaction logic to jointly map dual-modal interfaces and guide rational RNA sequence design without structural dependencies. Even though generalized biological foundation models like LucaOne [26] have shown an emergent understanding of principles across protein and nucleic acid sequences, a sequence-only model specialized for protein–RNA complexes is still urgently needed.

These limitations motivate a unified framework that integrates prediction, interpretation, and generation from sequences alone, capturing the motif-centric logic of protein–RNA complexes without structural dependencies. Such a model learns contextual and relational features associated with protein–RNA binding affinity and interfaces, and protein-binding RNA design, facilitating interactome modeling for data-sparse RBPs and motif-guided protein-binding RNA design. Previous studies, such as the template-based SPOT-Seq [27] with only protein sequences as the input, demonstrated the feasibility of coupling binding sites in proteins and affinity prediction through structural modeling. However, they only focus on protein–RNA interaction prediction, and do not simultaneously address binding prediction and sequence design.

In this study, we introduce ProRB, a unified sequence-based framework that jointly tackles three associated protein–RNA tasks: binding affinity prediction, dual-modal interface mapping on proteins and RNAs, and conditional *de novo* protein-binding RNA sequence generation, from paired sequences alone. Unlike prior isolated approaches, ProRB innovatively fuses protein and RNA embeddings through adaptive cross-modal attention without any 3D supervision. By sharing a joint embedding space, ProRB captures relational inductive biases where interface mapping and motif recognition provide mutual supervision for binding affinity estimation and sequence generation plausibility. To enable robust cross-modal alignment, we devise a novel dual self-supervised pretraining scheme: (i) information alignment via Kullback–Leibler (KL) divergence between protein-conditioned RNA representations and native embeddings, and (ii) masked language modeling on dynamically fused representations. A shared Transformer decoder then refines joint embeddings for task-specific predictions while enabling autoregressive, motif-preserving RNA synthesis conditioned on target proteins. Remarkably, ProRB sets new state-of-the-art benchmarks on the binding affinity and interface prediction, outperforming both sequence- and structure-based baselines. Its cross-attention maps further uncover interpretable, motif-centric binding logic that aligns well with experimentally validated motifs, empowering *de novo* protein-binding RNA designs with biophysical plausibility consistent with native RNAs and functional enrichment, serving as computationally prioritized candidate RNAs for further wet-lab validation. By seamlessly integrating prediction, interpretation, and generation in a single scalable framework, ProRB provides a new paradigm that facilitates motif-aware RNA therapeutic engineering.

## Materials and methods

### Benchmark datasets

For pretraining, we adopt the PRI30K dataset from CoPRA [5], which consists of non-redundant protein–RNA interacting pairs curated from the BioLiP2 database [28]. This dataset was constructed by extracting the maximum connected subgraph from 5 909 PDB complexes to address structural redundancy caused by symmetric assemblies. To ensure the computational efficiency and avoid training instability caused by super-long chains and redundant symmetric assemblies, the dataset only keeps protein chains with ${{L}_{\mathrm{protein}}} \le 750\,\mathrm{ aa}$ and RNA chains with $5 \le \ {{L}_r} \le 500$ nt. Finally, the pretraining dataset consists of 30 006 protein–RNA sequence pairs.

Binding affinity prediction is evaluated on PRA201, a high-quality protein–RNA binding affinity benchmark with 201 single sequence pairs curated from PDBbind [29], PRBABv2 [20], and ProNAB [30]. To ensure a rigorous evaluation, we employed a two-tiered clustering strategy for reducing data redundancy. First, for purely sequence-based filtering, protein sequences were clustered using PSI-CD-HIT [31] at stringent sequence identity thresholds of 20% and 25%. Furthermore, to group proteins based on structural similarity, we utilized Foldseek [32] to cluster the protein structures at thresholds of 40% and 50%. Cluster-based train–test splits with a ratio of 4:1 are generated across five random seeds for each of these sequence and structural similarity configurations. Experimental ∆*G* (kcal/mol) serves as the regression target.

Binding site prediction utilizes Q-BioLip [33] as the primary benchmark dataset. To ensure the structural integrity, we filtered complexes based on the sequence length (RNA: 5–510 nt; protein: 10–1022 aa) and excluded samples where the proportion of missing residues or nucleotides exceeded 30%. Additionally, the entries with an RNA minimum free energy (MFE) >−1 kcal/mol (calculated via ViennaRNA [34]) were removed to filter out unstable conformations. An interaction is defined if the minimum distance between any heavy atoms of a residue–nucleotide pair is less than the sum of their van der Waals radii plus 0.5 Å. Furthermore, in cases where identical protein and RNA sequence pairs were associated with differing binding site annotations, the interaction labels were merged to represent the union of all identified binding sites. To systematically investigate the influence of sequence redundancy on the model performance and generalization, protein sequences were clustered using CD-HIT [31] across a wide range of identity thresholds: 40%, 50%, 60%, 70%, 80%, and 90%. For each threshold, cluster representatives were selected to form the final dataset, resulting in exactly 821, 951, 1102, 1203, 1314, and 1448 unique protein sequence clusters, respectively. We employed a cluster-based split for training and test sets with a 4:1 ratio across five random seeds, ensuring that no sequence clusters overlapped between the training and test sets. Detailed statistical distributions of unique clusters, training samples, and test samples across all thresholds are shown in Supplementary Fig. S1.

To rigorously assess the generalization performance of ProRB against the state-of-the-art AF3 [24], we employed the recently established FoldBench dataset [35]. FoldBench is a comprehensive, low-homology benchmark with biological assemblies curated specifically to address the challenge of data leakage in all-atom structural predictors, with the data collection after November 2024. This choice is primarily motivated by preventing potential data leakage: as an all-atom foundation model trained on nearly the entire structural database, AF3 may have already encountered many samples within our curated training sets. Moreover, the stringent redundancy removal protocol employed by FoldBench ensures a fairer comparison, evaluating AF3 in a low-homology regime, where its heavy reliance on multiple sequence alignment (MSA) and co-evolutionary signals is naturally constrained, thereby providing a more objective benchmark for ProRB. Focusing on protein–RNA interfaces, we initially extracted all 70 protein–RNA single-chain complexes from FoldBench. To ensure an unbiased comparison, we implemented a multi-stage filtering protocol: (i) removing six samples that overlapped with the ProRB training set; (ii) applying consistent biophysical filters (RNA length 5–510 nt, protein length 10–1022 aa, and RNA MFE <−1 kcal/mol) as used in the Q-BioLip benchmark; and (iii) performing CD-HIT clustering at a 40% identity threshold to select only the cluster representatives. This process yielded a final subset of nine high-quality, highly divergent test cases. This subset represents the stringent evaluation, providing a unique platform for an unbiased performance comparison between ProRB and AF3.

For protein-binding RNA generation, the pretrained decoder is fine-tuned on the same binding site training set, with proteins clustered using CD-HIT at 70% identity; the performance of protein-binding RNA generation is evaluated on the binding site test set.

### Workflow of ProRB framework

**Figure 1.**
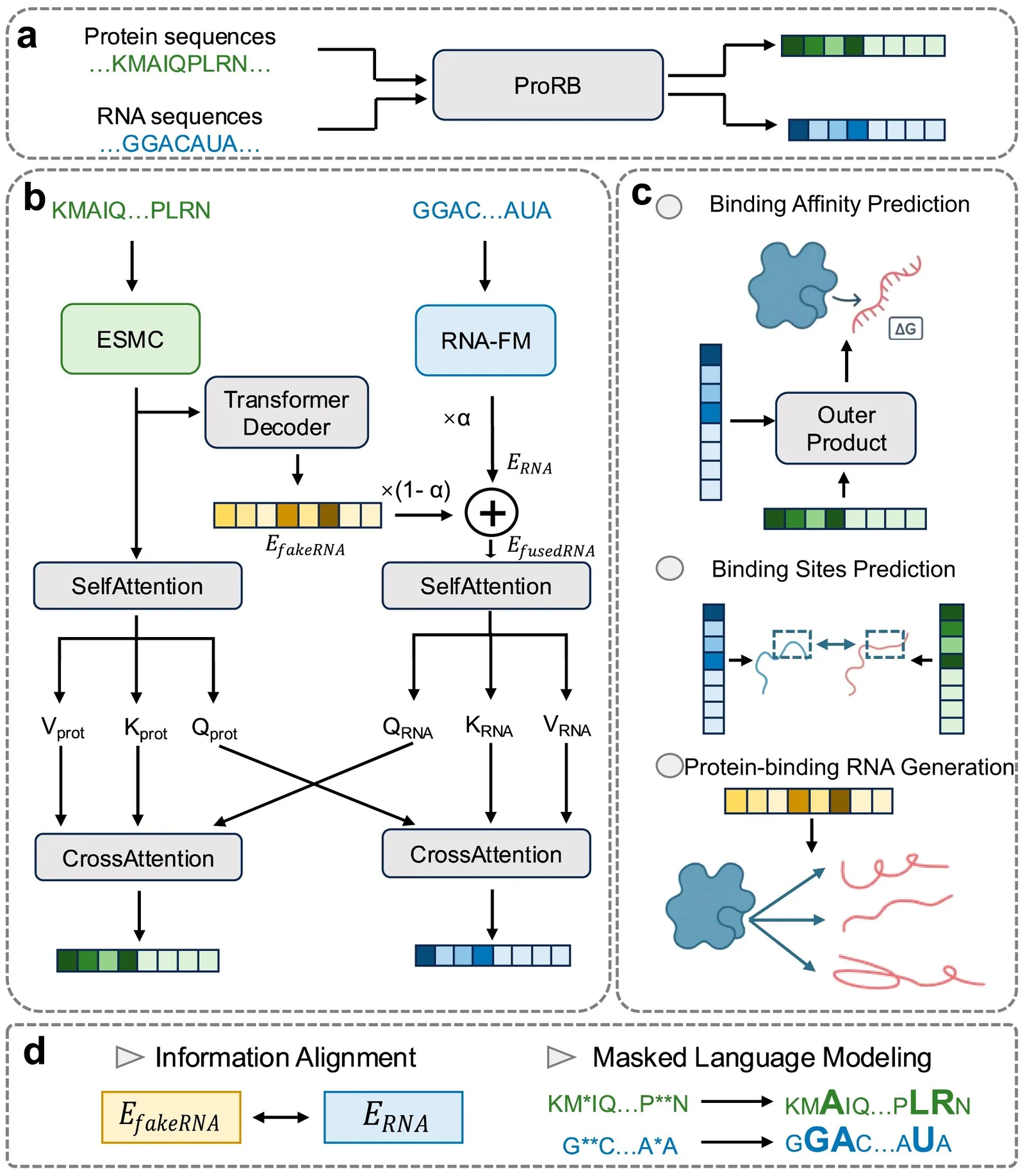
Overview of ProRB model. (**a**) ProRB is a unified, sequence-only foundation framework for protein–RNA interaction analysis. (**b**) Protein and RNA sequences are independently encoded by ESMC and RNA-FM, followed by adaptive cross-modal fusion weighted by a controllable coefficient α. Self-attention layers refine intra-modal representations, while cross-attention layers model inter-modal dependencies to generate aligned joint embeddings. (**c**) Task-specific heads enable three tasks: protein–RNA binding affinity (Δ*G*) prediction via outer-product interaction and MLP regression; dual-modal (residue- and nucleotide-level) binding site prediction in proteins and RNAs via lightweight classifiers; and *de novo* protein-binding RNA generation conditioned on the target protein via a cross-attention decoder. (**d**) Two self-supervised pretraining objectives enhance the cross-modal alignment: (i) information alignment, minimizing KL divergence between RNA embeddings from RNA-FM and generated by the Transformer decoder, which uses protein embeddings from ESMC as the memory input and RNA embeddings from RNA-FM as the target, and (ii) dual-modal masked language modeling, reconstructing masked tokens in both protein and RNA sequences.

ProRB is a unified, sequence-only transformer-based framework for joint modeling of protein–RNA binding recognition and *de novo* protein-binding RNA sequence generation. Protein and RNA sequences are independently encoded by ProRB to get respective embeddings (Fig. 1a). Protein and RNA sequences are first independently encoded using the protein language model ESMC [36] and the RNA language model RNA-FM [37], respectively, to obtain embeddings that incorporate evolutionary and structural priors directly from the sequences. The protein embeddings are then fed into a Transformer decoder as the decoder’s memory input, with the RNA embeddings serving as the target input, to produce a jointly learned RNA representation that encodes information from both modalities. Then, the two RNA embeddings are adaptively fused via a controllable weight α, as shown in Fig. 1b, enabling weighted cross-modal integration. Following this fusion, ProRB employs a dual-stream attention mechanism. Self-attention layers first refine the intra-modal sequence context for each modality, while cross-attention layers explicitly model the inter-modal residue–nucleotide interactions to generate aligned joint embeddings. These informative representations are then passed to three specialized functional heads: an outer-product interaction-aware regressor, which explicitly models the pairwise correlations between multimodal features by computing the outer product of global protein and RNA embeddings for protein–RNA binding affinity prediction, parallel lightweight classifiers for identifying residue- and nucleotide-level binding sites, and a cross-attention decoder for the autoregressive generation of *de novo* protein-binding RNAs (Fig. 1c). To strengthen the representation learning, we introduce two self-supervised pretraining objectives (Fig. 1d): (i) information alignment, minimizing the KL divergence between RNA embeddings from RNA-FM and generated by the decoder to enhance the cross-modal compatibility before fusion, the alignment also conditions RNA generation on specific proteins; and (ii) dual-modal masked language modeling (MLM) on fused embeddings, jointly reconstructing masked tokens from both modalities. Specifically, the MLM objective utilizes the native vocabularies of the foundation models, where the protein branch follows the ESMC tokenization scheme (Supplementary Table S1) and the RNA branch employs the vocabulary derived from RNA-FM (Supplementary Table S2). This end-to-end design establishes ProRB as a unified structure-free foundation framework for RNA–protein interactome, integrating protein–RNA binding affinity prediction, binding site interpretability, and conditional RNA design.

#### Embedding layers

To represent the primary sequences, we utilized the ESMC and RNA**-**FM foundation models to extract high-dimensional evolutionary and structural embeddings for proteins (${{d}_{\mathrm{protein}}} = 960$) and RNAs (${{d}_{\mathrm{ RNA}}} = 640$), respectively. To facilitate multi-modal integration, we employed modality-specific feedforward layers to project these embeddings into 64-dimensional latent spaces, ensuring dimensional alignment prior to subsequent feature fusion and yielding the embeddings ${{E}_{\mathrm{protein}}}$ and ${{E}_{\mathrm{ RNA}}}$ for subsequent feature fusion.

#### Feature projection layers

To facilitate synergistic integration of multi-modal features and enable protein-conditioned RNA sequence design, we designed a cross-modal projection module based on a Transformer decoder. This architecture serves as a functional bridge to map protein embeddings into the RNA representation space (Fig. 1b). The feature projection is formally defined as


(1) \begin{eqnarray*}
\begin{array}{*{20}{c}} {{{E}_{\mathrm{fakeRNA}}} = \ \mathrm{Decoder}\left( {\mathrm{memory} = {{E}_{\mathrm{protein}}},\ \mathrm{target} = {{E}_{\mathrm{ RNA}}}} \right) } \end{array},
\end{eqnarray*}

where Decoder denotes a standard Transformer architecture with two layers and four attention heads. This module transforms the initial protein-informed context into a synthetic RNA representation, ${{E}_{\mathrm{fakeRNA}}}$, which is subsequently used to integrate protein and RNA information.

Following this projection, we implement an adaptive fusion mechanism to integrate the original RNA-FM priors with the decoder-derived features. The two RNA representations are combined via a weighted sum, controlled by a fusion coefficient$\ \alpha $:


(2) \begin{eqnarray*}
\begin{array}{*{20}{c}} {{{E}_{\mathrm{fusedRNA}}} = \ \alpha {{E}_{\mathrm{ RNA}}} + \left( {1 - \alpha } \right){{E}_{\mathrm{fakeRNA}}}} .\end{array}
\end{eqnarray*}

This adaptive integration allows ProRB to dynamically balance the intrinsic structural information from RNA-FM with the cross-modal contextual features learned from protein sequence.

#### Feature fusion layers

Following the initial projection and alignment, ProRB employs a dual-stream attention mechanism to capture both intra-modal contexts and inter-modal dependencies. As illustrated in Fig. 1b, the protein representation ${{E}_{\mathrm{protein}}}$ and the fused RNA representation ${{E}_{\mathrm{fusedRNA}}}$ were first processed through independent self-attention layers comprising eight heads to refine their respective intra-modal sequence features.

Subsequently, to model the intricate binding logic between the two modalities, these refined embeddings are integrated via eight-head cross-attention layers. In this stage, the representations from one modality serve as the Query, while the other modality provides the Key and Value matrices. Thus, the protein features are updated by attending to the RNA sequence, and vice versa, enabling the model to learn co-evolutionary and spatially aware patterns characteristic of protein–RNA interfaces.

Finally, this dual-stream interaction yields the updated joint representations, denoted as ${{\tilde{E}}_{\mathrm{protein}}}$ and ${{\tilde{E}}_{\mathrm{ RNA}}}$, which encapsulate the mutually informed contextual features from both modalities.

#### Functional heads for protein–RNA binding affinity estimation and interface mapping

As illustrated in Fig. 1c, ProRB utilizes the updated joint representations to perform downstream prediction tasks through specialized functional heads.

For the protein–RNA binding affinity prediction task, the model integrates features from both modalities using an interaction-aware mechanism. In this approach, we compute the outer product of ${{\tilde{E}}_{\mathrm{protein}}}$ and ${{\tilde{E}}_{\mathrm{ RNA}}}$ to capture the comprehensive residue–nucleotide interaction landscape: $\mathrm{ Interaction}\ \mathrm{Matrix}\ = {{\tilde{E}}_{\mathrm{protein}}}\tilde{E}_{\mathrm{ RNA}}^T$. Subsequently, a summation is performed over the non-padded portions of the resulting interaction matrix to derive a global representation, which is then fed into a multi-layer perceptron (MLP) regressor to predict the protein–RNA binding free energy ${\mathrm{\Delta }}G$.

To identify the binding interface at the residue and nucleotide level, ProRB performs dual-modal interface mapping in parallel. We employ a shared lightweight linear classification head to process the final fused embeddings of both modalities, generating logit scores for each position. These scores are subsequently transformed into binding probabilities via a sigmoid function, allowing for direct comparison against ground-truth binary (0/1) interface labels. By utilizing a shared head, the model is encouraged to learn a unified representation space for binding sites, where both protein residues and RNA nucleotides are mapped to a consistent interaction probability scale.

#### Decoder training protocol

The Transformer-based decoder facilitates the alignment between protein and RNA representation spaces to enable cross-modal integration.

During pretraining, the decoder maps ESMC-derived protein representations to the RNA representation from RNA-FM. To bridge the gap between the two modalities, we introduce an information alignment objective. This is enforced via a KL divergence loss between the decoder’s output ${{E}_{\mathrm{fakeRNA}}}$ and the native RNA-FM embeddings ${{E}_{\mathrm{ RNA}}}$:


(3) \begin{eqnarray*}
\begin{array}{*{20}{c}} {{{L}_{\mathrm{ KL}}} = \mathrm{ KL}\left[ {\mathrm{softmax}\left( {\frac{{{{E}_{\mathrm{fakeRNA}}}}}{\tau }} \right)\|\mathrm{ softmax}\left( {\frac{{{{E}_{\mathrm{ RNA}}}}}{\tau }} \right)\ } \right] } \end{array},
\end{eqnarray*}

where the temperature *τ * is set to 0.8.

For *de novo* protein-binding RNA sequence design, the pretrained decoder is repurposed for autoregressive generation. Since the bidirectional nature of RNA-FM is ill-suited for sequential generation, we add three specialized layers during fine-tuning to implement the autoregressive feedback loop:

1. Token Projector (TP): an MLP that maps the decoder’s output ${{E}_{\mathrm{fakeRNA}}}$ to logits across the RNA-FM vocabulary (size = 26), enabling probabilistic nucleotide sampling at each step.
2. Embedding Projector (EP): an embedding layer that encodes the discrete sampled tokens back into a continuous latent space, which are subsequently augmented with positional encodings to preserve the sequential order of the generated RNA.
3. Causal Encoder Layer (CE): a transformer encoder layer equipped with a masked self-attention mechanism. It processes the positionally-enriched embeddings to model the historical context of the accumulating generated sequence, effectively transforming them into the target input required by the decoder for the subsequent step.

During fine-tuning, the decoder generates ${{E}_{\mathrm{fakeRNA}}}$ via a conditional autoregressive strategy. Although the native RNA-FM vocabulary already includes an end-of-sequence token, the original bidirectional representations learned by RNA-FM are inherently contextual and lack the directional constraints required for causal autoregressive decoding. To enforce prompt and precise termination during causal inference, we adjusted the loss function by up-weighting the cross-entropy loss of the existing < eos > token five-fold. This re-weighting strategy penalizes premature or delayed termination, compensating for the transition from bidirectional representation to unidirectional autoregressive generation without altering the native vocabulary size. In each iteration, the latent representation output by the decoder is mapped to the RNA-FM vocabulary space through the TP to yield nucleotide logits. Once a discrete token is sampled, it is re-embedded into the continuous space by the EP, followed by the CE layer, where the masked self-attention mechanism updates the accumulated historical context. The resulting representations are cycled back as the decoder’s target input for predicting the logits at next sequence position. The training objective function minimizes the cross-entropy loss between the predicted token probability distributions derived from the TP-generated ${{E}_{\mathrm{fakeRNA}}}$ and the ground-truth RNA indices derived from the RNA-FM tokenizer.

Notably, the native vocabulary of the RNA-FM foundation model consists of 26 tokens, encompassing canonical bases, degenerate IUPAC nucleotide codes (e.g. N, R, Y), and special tokens. During the fine-tuning phase under teacher forcing, we employ a seven-token logit mask, which permits the four canonical bases, the termination token < eos >, and two necessary boundary tokens < cls > and < mask > . Retaining < cls > and < mask > during training is essential for computing proper cross-entropy losses over tokenized sequence boundaries and maintaining structural alignment with RNA-FM’s native pre-trained representation, while degenerate bases and other unneeded tokens are effectively masked out. Importantly, during this fine-tuning stage, the model is trained using a teacher-forcing protocol where the inputs are the ground-truth sequence prefixes. Consequently, token-selection sampling is not active during training, rather strictly employed during the subsequent autoregressive inference phase. The workflow of the training and inference phases for protein-binding RNA sequence generation are shown in Supplementary Fig. S2.

### The large language models for input embedding in ProRB

To enhance the representational capacity, ProRB leverages foundation models for proteins and RNAs as the initial embedding layers, rather than one-hot encodings or other hand-designed representations. These foundation models are widely recognized for their ability to encode evolutionary signals and implicitly capture secondary and tertiary structural propensities. By incorporating such pre-trained embeddings, ProRB can initially encode key biophysical information, such as conserved motifs and binding determinants that underpin protein–RNA interactions, thereby boosting downstream task performance.

Among the various protein and RNA foundation models, we selected ESMC [36] and RNA-FM [37] to encode protein sequences and RNA sequences, respectively. For proteins, ESMC, an embedding model derived from the ESM3 framework, which fuses sequence, structural, and functional annotation data into a unified representation, enables the learning of multimodal associations and a holistic view of protein features. ESM3 has demonstrated strong efficacy in sequence design and structure prediction [36]. For RNAs, RNA-FM achieves promising performance in structural prediction tasks (e.g. secondary structure prediction) as well as functional annotation tasks (e.g. modification prediction and non-coding RNA function classification).

### Strategy of protein-binding RNA sequence generation

To enable *de novo* protein-binding RNA sequence design, ProRB employs a conditional autoregressive generation strategy during the inference phase. While the token vocabulary and the downstream prediction head theoretically output scores over all tokens in RNA-FM’s vocabulary, the actual generation loop is executed with explicit, step-wise token-selection controls to balance the sequence diversity and biophysical constraints.

Specifically, at each autoregressive step, the token selection proceeds through the following processes:

1. Logit masking: To prevent the generation of non-canonical bases, degenerate nucleotides, or structural artifacts, a hard logit mask is applied to the projected vocabulary logits ${{z}_t}$ produced by the Token Projector (TP). This assigns a logit value of * - ∞ * to all non-eligible tokens, restricting the active selection pool to the four canonical bases {A, U, G, C} and the designated End-of-Sequence token < eos>:
  (4) \begin{equation*}
\begin{array}{@{}*{1}{c}@{}} {z_t^{\prime} = {{z}_t} + \textit{Mask},\ \textit{where}\ mas{{k}_i} = \left\{ {\begin{array}{@{}*{1}{c}@{}} {0,\ i \in \left\{ {A,\ U,\ G,\ C,\ \left\langle {eos} \right\rangle } \right\}}\\ { - \infty ,\ \textit{otherwise}} \end{array}} \right. } \end{array}
\end{equation*}
2. Temperature scaling and probability assignment: The masked logits $z_t^{\prime}$ are scaled by a sampling temperature T before executing the Softmax transformation to derive the step-wise probability distribution:
  (5) \begin{eqnarray*}
\mathrm{ logits} = \mathrm{ Softmax}\left( {\frac{{z_t^{\prime}}}{T}}\right) .
\end{eqnarray*}
3. Top-k filtering and multinomial sampling: Instead of executing a deterministic greedy search, a top-k filter (k = 4) is applied to extract the k most probable eligible tokens. The filtered probabilities are then normalized, and the final token is sampled via a multinomial distribution to inject controlled stochastic diversity:

(6) \begin{eqnarray*}
&&\mathrm{token}\sim \mathrm{ Multinomial}\left[ \mathrm{ Top} - 4\left( {\mathrm{logits}} \right] \right),\\&&\quad \mathrm{where}\ \mathrm{token} \in\left\{ \mathrm{ A},\ \mathrm{ U},\ \mathrm{ G},\ \mathrm{ C},\ \left\langle {\mathrm{ eos}} \right\rangle \right\}.
\end{eqnarray*}

This rigorous masking and sampling scheme guarantees that no non-canonical bases or artifact tokens can be sampled or generated during inference, ensuring the biophysical validity of the designed RNA sequences.

Once a discrete token is sampled, it is appended to the historical sequence. The entire accumulated sequence of generated tokens is then mapped to a continuous latent space via the EP and updated through the CE layer to refresh the sequence context. The resulting representations are fed as the decoder’s target input for predicting the next sequence position, effectively driving sequential RNA generation conditioned on the protein embeddings. This mechanism ensures the progressive integration of binding-compatible features into the generated sequence.

To maintain biological fidelity and prevent the accumulation of non-canonical sequences, the generation process strictly monitors the occurrence of the < eos > token. The generation process continues iteratively if a canonical base {A, U, G, C} is selected, but will terminate immediately if the token matches the < eos > token. This mechanism ensures that the generated RNA candidates can span a wide range of lengths and structural motifs, thereby capturing the inherent functional diversity of the protein–RNA interactome compared to fixed-length or heuristic-based approaches. Furthermore, the specific combination of nucleotides is controlled by the adaptive cross-modal attention, which prioritizes the synthesis of high-affinity motifs (such as RNA recognition motif-binding sequences) while maintaining global biophysical constraints like GC content and folding stability.

The temperature parameter *T* is introduced during the Softmax transformation of the projected vectors. By scaling the logits before the final probability assignment, *T* modulates the balance between the generative diversity and structural fidelity: lower values of *T* sharpen the distribution toward high-confidence nucleotides, while higher values encourage the exploration of the sequence space. In our framework, we systematically evaluated temperatures ranging from 0.4 to 1.6. Empirically, we selected *T = 0.8* as the default sampling temperature to make an optimal balance between sequence diversity, which will collapse at *T ≤ 0.6*, and sequence fluency plus motif fidelity, which will degrade into random-like sequences at *T ≥ 1.2*.

To evaluate the fluency and biological plausibility of the generated RNA sequences, we utilize perplexity (PPL) as a key scoring metric. The perplexity for each candidate sequence is calculated based on the probability distribution output by the Transformer decoder during the autoregressive generation process.

Formally, for a generated RNA sequence of length *N*, the perplexity is defined as the exponential of the negative average log-probability per functional nucleotide:


(7) \begin{eqnarray*}
\mathrm{ PPL} = {\mathrm{exp}}\, ( - \frac{1}{N}\mathop \sum \limits_{i = 1}^N \mathrm{log}\textit{P}\left( {{{x}_i}{\mathrm{|}}{{x}_{ < i}},\ {{E}_{\mathrm{protein}}}} \right))\ ,
\end{eqnarray*}

where $P( {{{x}_i}{\mathrm{|}}{{x}_{ < i}},\ {{E}_{\mathrm{protein}}}} )$ represents the conditional probability of the *i*th sampled nucleotide given the preceding sequence and the protein memory.

Consequently, candidate sequences undergo a multi-stage filtering and ranking protocol to prioritize high-fluency, biochemically viable designs. First, sequences with infinite perplexity are discarded, these instances represent immediate-termination artifacts where the < eos > token is sampled at the very first autoregressive step, resulting in null sequences of zero active length. Eliminating these computational artifacts is necessary to accurately evaluate the actual length preferences of the generator. Second, to filter out biophysically unstable RNAs, candidate RNAs with an MFE >−1 kcal/mol are excluded. We note that this thermodynamic filter does not introduce downstream length selection bias, as the length distribution of valid sequences remains highly consistent before and after the MFE filtering. Although such short or unstructured oligonucleotides are occasionally documented within co-crystallized complexes in the PDB, they fundamentally lack independent thermodynamic stability under physiological conditions and are prone to random coil degradation.

Finally, the remaining sequences are prioritized using a composite ranking score S_rank_ that systematically balances the sequence fluency and thermodynamic binding affinity, formally defined as


(8) \begin{eqnarray*}
\begin{array}{*{20}{c}} {{{S}_{\mathrm{rank}}} = w1 \times \mathrm{ PPL} + w2 \times \triangle {{G}_{\mathrm{pred}}}} \end{array},
\end{eqnarray*}

where $\Delta {{G}_{\mathrm{pred}}}$ denotes the ProRB-predicted binding free energy and *w1* and *w2* are the combination weight.

To ensure the physical relevance of this sequence-level scoring function, the coefficients *w1* and *w2* were calibrated via linear regression on a pilot calibration subset, optimizing the weights to maximize the alignment with explicit 3D structural docking confidence evaluated by AF3 interface predicted TM-score (ipTM). The integrated filtering and ranking mechanism allow ProRB to effectively eliminate generation artifacts and systematically enrich robust, motif-preserving RNA candidates.

### Performance metrics

To evaluate ProRB’s performance across three tasks, we employed standard metrics tailored to each task, ensuring the comparability with prior work. All prediction related metrics were computed on held-out test sets, with results reported as mean ± standard deviation over five random seeds for robustness.

#### Protein–RNA binding affinity prediction

Binding free energy (Δ*G*, in kcal/mol) was predicted as a regression task, ${{y}_i}$ is true binding free energy, and ${{\hat{y}}_i}$ is predicted binding free energy:

(1) Root mean squared error (RMSE): $\sqrt {\frac{1}{n}\mathop \sum \limits_{i = 1}^n \ {{{({{y}_i} - {{{\hat{y}}}_i})}}^2}} $, measuring absolute prediction error.

(2) Mean absolute error (MAE): $\frac{1}{n}\mathop \sum \limits_{i = 1}^n \mid {{y}_i} - {{\hat{y}}_i}\mid $, assessing average deviation.

(3) Pearson correlation coefficient (r): $\frac{\Sigma(y_i-\grave{y})(\hat{y}_{i}-\overset{\prime}{\hat{y}})}{\sqrt{{\Sigma} (y_i-{\grave{y}})^{2}(\hat{y_i}-\overset{\prime}{\hat{y}})^{2}}} $, evaluating linear agreement.

(4) Spearman’s rank correlation (ρ): non-parametric measure of monotonic relationship, computed via Kendall’s tau approximation.

#### Protein–RNA binding site prediction

Binding residues/nucleotides were predicted as binary classification per position (binding: 1; non-binding: 0). Due to the class imbalance (positive rates ∼1%–5%), we calculated the area under precision-recall curve (AUPRC) and area under receiver operating characteristic curve (AUROC)

#### Protein-binding RNA sequence generation

The generated RNA sequences were evaluated through a hierarchical framework encompassing distributional fidelity, biophysical plausibility, and complex-aware functional relevance:

(1) Length distribution (fidelity check): distribution of sequences’ lengths.

(2) GC content (nucleotide composition): fraction of G/C bases.

(3) MFE (kcal/mol, structural foldability): it is computed with ViennaRNA [34]; more negative values denote better stability.

Structural plausibility is evaluated using AF3 [24].

(4) Predicted TM-score (pTM): 0–1 scale for global fold confidence.

(5) Interface pTM (ipTM): focused on the protein–RNA binding sites.

#### Motif enrichment

We use the perplexity to filter-generated protein-binding RNA sequences (finite, low values prioritized for fluency), then the filtered sequences are scanned with FIMO [38] (*q* < 0.001 threshold) against RNAcompete motifs [23].

These metrics collectively quantify the predictive accuracy, interpretability, and generative utility, with thresholds aligned to benchmarks.

### Baseline methods

#### Baseline methods for protein–RNA binding affinity prediction

(1) DeePNAP (sequence-only CNN): DeePNAP utilizes a 1D-convolutional neural network (1D-CNN) architecture comprising four parallel branches with different receptive fields to extract features from protein and RNA sequences, including combinatorial encoding and the categorization of side-chain physical properties (e.g. polarity, charge, and hydrophobicity).

(2) CoPRA (structure-informed foundation model): CoPRA represents the current state-of-the-art structure-based approach that bridges cross-domain pre-trained language models (ESM-2 for proteins and RiNALMo for RNAs) with 3D complex structures. It employs a Co-Former architecture to fuse sequence embeddings with pairwise structural features (distance and angular information) extracted from the binding interface.

(3) OmniBioTE (multi-omic foundation model): OmniBioTE is a large-scale multi-omic biosequence Transformer pretrained on over 250 billion tokens comprising mixed protein (UniRef100) and nucleic acid (GenBank) sequence data. It utilizes a non-causal encoder Transformer architecture based on LLaMA-2 to emergently learn joint, modality-invariant representations between proteins and nucleic acids through a masked language modeling objective. For the binding affinity prediction task, the model is fine-tuned to regress the change in Gibbs free energy from concatenated protein–RNA sequence pairs followed by a task-specific linear projection.

#### Baseline methods for protein–RNA binding site prediction

Frame Averaging (FA) Transformer: Frame Averaging (FAFormer) is a structure-based method designed for Protein–nucleic acid complex modeling. The core of FA lies in its use of Frame Averaging to achieve Euclidean symmetry [E(3)-invariance/equivariance] without the high computational cost of specialized equivariant kernels. Specifically, FA constructs local coordinate systems (frames) for each residue or nucleotide and averages the model’s outputs over these frames to ensure that the predicted binding sites are independent of the complex’s rotation or translation.

### AlphaFold 3

AF3 is a state-of-the-art all-atom structure prediction framework designed to unify the modeling of proteins, nucleic acids, ligands, and ions. In the binding site prediction task, AF3 provides high-resolution complex structures, enabling the identification of interaction interfaces based on atomic distance thresholds. Due to local hardware and GPU memory limitations in deploying the massive, full-scale AF3 pipeline locally, all AF3 predictions in this study were retrieved using the official web-based AlphaFold Server under default parameter settings.

#### Baseline methods for protein-binding RNA generation

Random control generation: to provide a rigorous baseline for biophysical property comparisons, we generated a set of length-matched random RNA sequences. For each ProRB-designed candidate, a corresponding random control of the same length was produced by stochastically sampling nucleotides from a uniform distribution. This empirical sampling approach ensures that the control group accounts for the same length-dependent variance as the generative designs.

#### Ablation variants of ProRB

To validate the necessity of the unified framework, we compared the full ProRB model against several variants:

(1) w/o self-attn: models without self-attention layers to isolate the importance of intra-modal contextual refinement.

(2) w/o cross-attn: models utilizing simple concatenation instead of cross-modal attention for inter-modal relational learning.

(3) *α *-variants: models with varying coefficients ($\alpha = \ \{ {0.0,\ 0.2,\ 0.4,\ 0.6,\ 0.8,\ 1.0} \}$) to determine the optimal balance between RNA-FM priors and protein-conditioned decoder features.

### Experimental setting

#### Hyperparameters and optimization

To ensure the reproducibility and eliminate the ambiguity, we clarify the multi-stage optimization protocols as follows:

(1) Pretraining phase: the model was optimized on the PRI30K dataset using the standard Adam optimizer with a learning rate of $1 \times {{10}^{ - 4}}$, ${{\beta }_1} = 0.9$, ${{\beta }_2} = 0.999$, and $\epsilon = 1 \times {{10}^{ - 8}}$. The training was performed with a batch size of 16 for 40 epochs.

(2) Protein–RNA binding affinity & binding site prediction fine-tuning: we employed the AdamW optimizer with a learning rate of $1 \times {{10}^{ - 4}}$, a weight decay of 0.01, ${{\beta }_1} = 0.9$, and ${{\beta }_2} = 0.99$. The batch size was set to 16. For the protein–RNA binding affinity prediction task, we implemented an early stopping strategy with a patience of 20 epochs based on the validation loss to mitigate overfitting and ensure optimal convergence. For the protein–RNA binding site prediction, the models were trained for 100 epochs as monitored by their respective training objectives.

(3) Protein-binding RNA generation fine-tuning: to accommodate the causal autoregressive decoder’s optimization landscape, the model was fine-tuned using the AdamW optimizer with a learning rate of $4 \times {{10}^{ - 4}}$, coupled with a CosineAnnealingLR scheduler (minimum learning rate of $1 \times {{10}^{ - 6}}$, ${{T}_{\mathrm{ max}}} = 80$). A batch size of 8 was applied for 80 epochs.

In addition, *w1* and *w2* in the ranking score ${{S}_{\mathrm{rank}}}$ for designed sequences is respectively optimized as −0.11 and −0.15.

#### Training objectives and loss functions

The model was optimized through task-specific loss functions to handle the diverse requirements of the unified framework:

1. Pretraining: We utilized KL divergence loss for cross-modal information alignment and cross-entropy loss for the dual-modal MLM task. Following the standard BERT-style protocol, we implemented a 15% masking rate, where padding regions are excluded from the selection pool. Specifically, for the selected tokens, 80% are replaced with a special [MASK] token, 10% are replaced with a random token from the vocabulary, and the remaining 10% are kept unchanged. During loss calculation, all non-masked positions and padding tokens are assigned a target label of -100, which are completely bypassed via the cross-entropy loss with ignore_index=-100 to prevent any structural gradient leakage. This masking policy encourages the model to learn robust, context-aware representations across both protein and RNA modalities.
2. Binding affinity prediction: This regression task utilized a combination of RMSE and MSE losses to minimize the deviation between predicted and experimental binding free energy *Δ G*.
3. Binding site identification: For dual-modal interface mapping, we employed BCEWithLogitsLoss to supervise the binary classification of interaction probabilities for residues and nucleotides.
4. RNA sequence generation: The decoder was fine-tuned using cross-entropy loss to optimize the autoregressive generation of nucleotide sequences.

#### Computational resources

All model training and inference were conducted on NVIDIA RTX 4090 GPUs (24GB). The sequence-only nature of ProRB allows for high computational efficiency compared to geometry-heavy structure-based methods.

## Results

### ProRB achieves superior performance on protein–RNA binding affinity prediction

We first evaluated ProRB on the protein–RNA binding affinity benchmark dataset PRA201. To rigorously assess the model’s generalization capabilities under strict redundancy removal, the evaluation was conducted across stringent splits, including sequence identity thresholds of 20% and 25% via PSI-CD-HIT and structural similarity thresholds of 40% and 50% via Foldseek. We assessed the performance using four metrics: RMSE and MAE for error quantification; Pearson (r) and Spearman (ρ) correlations for linear and rank-based agreement with experimental Δ*G*.

**Figure 2.**
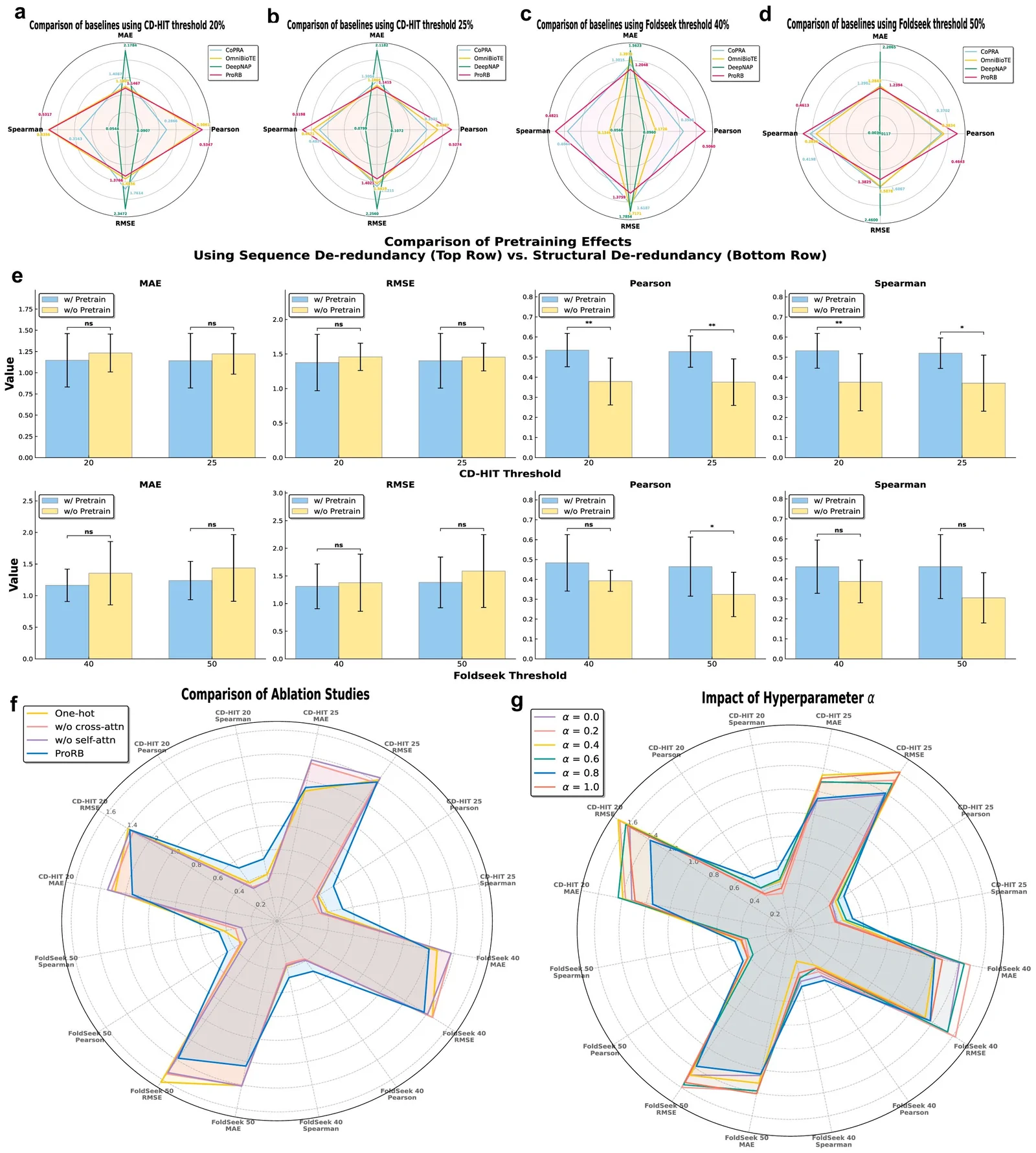
Performance of ProRB on the protein–RNA binding affinity prediction. Performance evaluation (RMSE, MAE, Pearson *r*, and Spearman *σ*) of ProRB compared with deep learning baselines (CoPRA, DeePNAP, and OmniBioTE) across diverse de-redundancy schemes, including highly stringent sequence similarity cutoffs at (**a**) 20% and (**b**) 25% via PSI-CD-HIT, and structural similarity cutoffs at (**c**) 40% and (**d**) 50% via Foldseek. Source data are provided in Supplementary Table S3. (**e**) Evaluation of pretraining effects under sequence de-redundancy (top row: 20% and 25% CD-HIT thresholds) versus structural de-redundancy (bottom row: 40% and 50% Foldseek thresholds), with statistical significance. Source data are provided in Supplementary Table S4. (**f**) Radar chart illustrating architectural ablation studies (w/o cross-attn, w/o self-attn, and Onehot baseline) evaluated across all sequence and structural thresholds. Source data are provided in Supplementary Table S5. (**g**) Sensitivity analysis mapping the impact of the hyperparameter fusion coefficient *α* ranging from 0.0 to 1.0. Source data are provided in Supplementary Table S6. All results are reported as mean values averaged over five random seeds, with standard deviations reported.

As shown in Fig. 2a–d, ProRB outperforms existing deep learning baselines, including the large-scale multi-omic foundation model OmniBioTE, the structure-based method CoPRA, and the CNN-based DeePNAP, across all sequence and structural identity thresholds. For instance, under the most rigorous sequence-based threshold 20%, ProRB yields an RMSE of *1.3766 ± 0.3649*, an MAE of *1.1467 ± 0.2816*, a Pearson *r* of *0.5347 ± 0.0741*, and a Spearman *ρ * of *0.5317 ± 0.0773*, consistently surpassing OmniBioTE across different splits. This advantage is particularly pronounced under the stringent Foldseek 40% structural clustering: ProRB reduces the RMSE by 19.9% (*1.3759 ± 0.3242* versus *1.7171 ± 0.1219* for OmniBioTE) and MAE by 13.8% (*1.2048 ± 0.2056* versus *1.3973 ± 0.1335* for OmniBioTE), while maintaining a robust Pearson correlation of *0.5060 ± 0.1138* compared to OmniBioTE’s sharp degradation to *0.1726 ± 0.1603*. Furthermore, ProRB substantially exceeds the performance of CoPRA, a structure-based method utilizing 3D atomic coordinates. At the 20% sequence threshold, ProRB reduces CoPRA’s RMSE (*1.7614 ± 0.2546*) by 21.8% and MAE (*1.4087 ± 0.2455*) by 18.6%, while nearly doubling the Pearson correlation (*0.5347 ± 0.0741* versus *0.2866 ± 0.1741*). Compared to the CNN-based DeePNAP (*2.3472 ± 1.1970* for RMSE and *2.1784 ± 1.2933* for MAE at the 20% sequence threshold), ProRB reduces the RMSE and MAE by 41.3% and 47.4%, respectively. To confirm the robustness of these improvements, the detailed statistical significance testing results for all baseline comparisons are provided in Supplementary Fig. S3. These findings collectively demonstrate that ProRB’s ability to uncover latent cross-modal patterns allows it to outperform both structure-aware and large-scale sequence models.

To systematically evaluate the contribution of our pretraining scheme to protein–RNA binding affinity prediction, we conducted a rigorous ablation analysis by comparing the full ProRB model against a variant without pretraining across all sequence and structural redundancy thresholds (Fig. 2e). For sequence-based redundancy removal at the highly stringent 20% identity threshold, the introduction of pretraining boosts the Pearson correlation by 41.3%, rising from *0.3785 ± 0.1166* to *0.5347 ± 0.0741*. Similarly, the Spearman correlation improves by 41.8%. This enhancement in correlation is consistently observed at the 25% identity threshold. Beyond correlations, pretraining consistently decreases absolute prediction errors at the 20% sequence threshold, reducing the mean MAE from *1.2323 ± 0.2227* to *1.1467 ± 0.2816* and the mean RMSE from *1.4588 ± 0.1973* to *1.3766 ± 0.3649*. This positive impact of pretraining remains robust when shifting to structural de-redundancy controls. Under the Foldseek 50% threshold, the pretrained ProRB model simultaneously reduces the MAE from *1.4383 ± 0.5273* to *1.2394 ± 0.2714* and the RMSE from *1.5880 ± 0.6586* to *1.3825 ± 0.4102*. Even at the most challenging threshold 40% of structural clustering, where homologous structures are strictly excluded, the pretrained model demonstrates superior error-reduction capabilities, yielding a lower MAE (*1.2048 ± 0.2056* versus *1.3551 ± 0.5020* without pretraining) and a lower RMSE (*1.3759 ± 0.3242* versus *1.3772 ± 0.5167*), while successfully improving the Pearson correlation from *0.3928 ± 0.0531* to *0.5060 ± 0.1138*. Taken together, our dual-modal pretraining successfully endows ProRB with a transferable and robust understanding of the complex biophysical rules governing protein–RNA binding affinities.

As shown in Fig. 2f, at the stringent sequence threshold 20%, the omission of cross-attention leads to a sharp decline in Pearson correlation (${\mathrm{\Delta }}r = - 0.1909$) and Spearman correlation (${\mathrm{\Delta }}\rho = - 0.1923$). This performance drop is even more pronounced at the structural threshold 50%, where the Pearson *r* decreases by *0.1189* and the MAE increases by *0.1667*. Similarly, removing the intra-modal self-attention layers, which refine the separate sequence contexts, also results in consistent performance drops. Under the sequence threshold 20%, removing self-attention decreases the Pearson *r* by *0.2048* and Spearman *ρ * by *0.1854*. Under structural threshold 50%, this ablation results in a degradation of ${\mathrm{\Delta }}r = - 0.1793$ and increases the prediction error (${\mathrm{\Delta MAE}} = + 0.1711$, ${\mathrm{\Delta RMSE}} = + 0.1451$). Lastly, substituting the foundation model representations with standard one-hot encodings leads to performance degradation across all evaluation scenarios. Under the sequence threshold 20%, the One-Hot baseline exhibits a drop of ${\mathrm{\Delta }}r = - 0.1493$ and an increase in absolute error (${\mathrm{\Delta MAE}} = + 0.1378$). At structural threshold 50%, using one-hot encoding leads to a dramatic error increase of ${\mathrm{\Delta RMSE}} = + 0.2416$ and a correlation loss of ${\mathrm{\Delta }}r = - 0.1372$. The results confirm that both the tailored attention mechanism and pretrained sequence-based evolutionary priors are essential for structure-free representation learning of protein–RNA complexes.

To investigate how to best balance the intrinsic structural information from the RNA-FM priors with the protein-conditioned contextual representations generated by the decoder, we systematically swept the fusion coefficient *α * from *0.0* to *1.0* (Fig. 2g). Across all sequence and structural redundancy thresholds, *α = 0.8* was empirically chosen as a balanced compromise, since it achieves consistently robust and competitive performance across various evaluation metrics and clustering thresholds. Setting *α = 1.0*, which completely ignores the decoder-enhanced contextual embeddings and relies purely on RNA-FM sequence representations, decreases the Pearson correlation by ${\mathrm{\Delta }}r = - 0.1588$ and increases the RMSE by * + 0.2241* under sequence threshold 20%. Under structural threshold 50%, *α = 1.0* yields a degradation of ${\mathrm{\Delta }}r = - 0.0435$ and an error increase of ${\mathrm{\Delta RMSE}} = + 0.1656$. Conversely, setting *α = 0.0*, which completely bypasses the native RNA-FM evolutionary embeddings and relies solely on the protein-derived RNA representation, also results in notable performance loss. At the sequence threshold 20%, the model with *α = 0.0* yields an increase in absolute error of ${\mathrm{\Delta MAE}} = + 0.1743$, a ${\mathrm{\Delta RMSE}}$ of * + 0.2016*, and a drop of ${\mathrm{\Delta }}r = - 0.1014$. Similarly, at the structural threshold 50%, *α = 0.0* degrades the Pearson correlation by ${\mathrm{\Delta }}r = - 0.0457$ and increases the error by ${\mathrm{\Delta RMSE}} = + 0.0904$. These findings justify the adaptive cross-modal integration design, demonstrating that while native embeddings from foundation models provide biological priors, the decoder-based protein-guided alignment is vital for tuning the joint embedding space to capture active interaction thermodynamics.

In summary, ProRB establishes a new sequence-based protein–RNA binding affinity prediction approach, driven by the integration of foundation models, attention mechanisms, a cross-modal alignment decoder, and a tailored pre-training strategy. Its high accuracy without requiring structural inputs enhances its wide applicability in RNA regulatory studies and RNA-targeted therapeutic design.

### ProRB outperforms existing methods on protein–RNA binding residues and nucleotides prediction

Accurate protein–RNA binding interface identification is crucial for understanding protein–RNA recognition. RNA–protein recognition is largely dictated by short linear motifs, which are inherently encoded along the 1D sequence[39–45]. Current approaches for binding site prediction are predominantly limited to single-modality analyses (e.g. protein-only [10, 11] or RNA-only [12] interfaces), which fail to capture knowledge specific to protein–RNA complexes.

ProRB employs a dual-modal approach for predicting binding probabilities of protein residues and RNA nucleotides independently. We evaluated ProRB for predicting binding residues and nucleotides on Q-BioLip [33] dataset and defined residue–nucleotide pair interaction if the minimum distance between any heavy atoms is smaller than the sum of van der Waals radii +0.5 Å [10].

**Figure 3.**
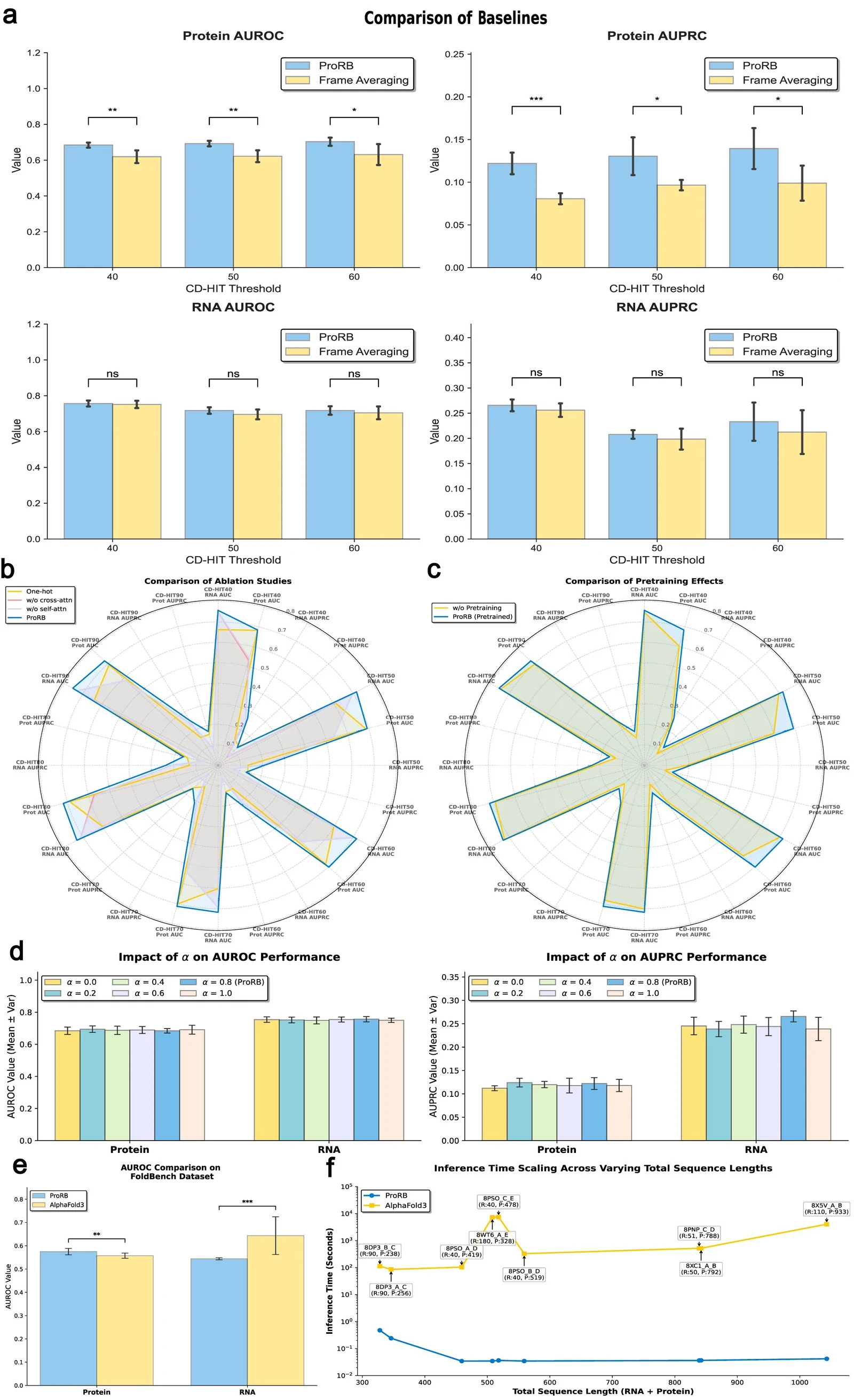
Dual-modal protein–RNA binding site prediction performance and ablation analysis on Q-BioLip of ProRB. (**a**) ProRB outperforms the structure-based method Frame Averaging in both binding residue and nucleotide prediction. Source data are provided in Supplementary Table S7. (**b**) Ablation studies of self-attention, cross-attention, and foundation model embeddings across various CD-HIT thresholds. The removal of embedding from foundation models, intra-modal self-attention, or inter-modal cross-attention results in the performance degradation. Source data are provided in Supplementary Table S8. (**c**) Assessment of the dual-modal pretraining scheme. The radar chart displays the performance gains (pretrained ProRB minus non-pretrained ProRB). Source data are provided in Supplementary Table S9. (**d**) Impact of the fusion coefficient *α * on AUROC and AUPRC performance at the 40% CD-HIT threshold. RNA interface recognition exhibits higher sensitivity to *α * than protein residues, with *α = 0.8* serving as an empirically selected compromise that performs well across different metrics. Source data are provided in Supplementary Table S10. (**e**) Performance on the low-homology FoldBench dataset compared to AF3. ProRB demonstrates interface recognition performance competitive with or superior to AF3 in protein binding residue prediction. (**f**) Computational efficiency comparison between ProRB and AF3. The inference time is plotted against varying total sequence lengths (RNA + protein), showcasing ProRB’s several-order-of-magnitude speed advantage. Results in panels (a), (d), and (e) are reported as mean * ± * standard deviation over five random seeds. Asterisks denote statistical significance (*, *P* < .05; **, *P* < .01; ***, *P* < .001; ns, non-significant).

We compare ProRB with the structure-based approach Frame Averaging (FA) method [17] (Fig. 3a) on the curated Q-BioLip dataset. Despite relying solely on sequence inputs, ProRB consistently outperforms FA, which utilizes 3D atomic coordinates and a geometric Transformer architecture, particularly in identifying binding interfaces on the protein side. For protein binding residue prediction, ProRB demonstrates the performance gains over FA across all CD-HIT clustering thresholds. Specifically, at a strict 40% identity threshold, ProRB achieves an AUROC of 0.684 ± 0.014 and an AUPRC of 0.122 ± 0.013 for protein binding interfaces, significantly higher than FA’s 0.619 ± 0.036 and 0.081 ± 0.007, respectively (Fig. 3a, top). Similar robust performance is observed at 50% and 60% thresholds, where ProRB maintains a clear advantage in capturing the sparse interaction signals on protein binding interfaces. Regarding nucleotide binding prediction on RNAs, ProRB yields competitive results compared to FA, achieving an AUROC of 0.757 ± 0.017 and an AUPRC of 0.266 ± 0.012 at the 40% threshold (Fig. 3a, bottom). While the performance on the RNA side is comparable to the structure-based baseline (marked as non-significant in Fig. 3a), the overall consistency across diverse identity levels underscores ProRB’s ability to capture structural interaction logic without structural dependencies. These results demonstrate that the enrichment of evolutionary priors from foundation models, combined with the adaptive cross-modal attention, allows ProRB to achieve superior dual-modal interface recognition, rivaling or surpassing geometry-aware structure-based models.

At the most stringent 40% CD-HIT identity threshold, ablation studies quantify the individual contributions of ProRB’s core components to dual-modal interface prediction. As shown in Fig. 3b, replacing the pretrained ESMC and RNA-FM embeddings with standard one-hot encoding severely impairs the model’s capacity to capture evolutionary and structural priors, which causes the RNA-side AUPRC to decrease from 0.266 to 0.152 and the AUROC to decrease from 0.757 to 0.662. Furthermore, the removal of self-attention layers responsible for refining intra-modal sequence contexts leads to a sharp decline in protein-side interface recognition, with the protein AUPRC dropping from 0.122 to 0.062. Moreover, deleting the cross-attention modules designed for explicit inter-modal residue–nucleotide modeling results in the most substantial performance degradation, reducing the protein AUPRC from 0.122 to 0.057 and the protein AUROC from 0.684 to 0.530. In summary, the results emphasize that while foundation models provide essential biological descriptors, ProRB’s dual-stream attention mechanism is indispensable for achieving high-precision interface mapping under low-similarity conditions.

As shown in Fig. 3c, the dual-modal pretraining scheme proves even effective at the stringent 40% CD-HIT identity threshold, where it significantly enhances the interface mapping performance by enabling ProRB to capture structural interaction logic through the self-supervised alignment. Specifically, the pretraining phase increases the protein AUPRC from 0.080 to 0.122, and boosts the protein AUROC from 0.603 to 0.684. The result demonstrates that the information alignment via KL divergence and masked language modeling helps the model effectively generalize to evolutionarily divergent protein families even when high-resolution structural templates are unavailable.

Then, we investigated the impact of the fusion coefficient *α *, which balances decoder-derived features with RNA-FM priors, across diverse sequence redundancy levels (Fig. 3d). While protein-side performance remains relatively robust to changes in the fusion weight, RNA interface recognition exhibits a distinct sensitivity at the 40% CD-HIT threshold, where the optimal alignment weight *α = 0.8* achieves a peak AUPRC of 0.266, outperforming both the *α = 0.0* baseline (0.245) and the *α = 1.0* pure-prior approach (0.239). The results confirm that for highly divergent targets, the balanced integration of intrinsic RNA evolutionary signals and protein-conditioned contextual features is essential for capturing the precise interaction logic and biophysical constraints of the protein–RNA interface.

To rigorously assess the generalization performance of ProRB against state-of-the-art all-atom structural predictors, we conducted a benchmark evaluation on the stringent, low-homology FoldBench dataset. AF3 was selected as the sole representative for this structural comparison, as it has been established as the top-performing model on this benchmark, serving as the gold-standard baseline for evaluating interface recognition. As shown in Fig. 3e, ProRB achieves the interface recognition performance that is competitive with AF3, despite being a structure-free model. On the subset, ProRB displays a marginal numerical advantage in protein-binding residue prediction (AUROC of 0.5749 versus 0.5574 for AF3), though the robustness of the difference remains highly sensitive to the sample scale. Conversely, AF3 outperforms ProRB in RNA binding nucleotide prediction (AUROC of 0.6434 versus 0.5441 of ProRB), demonstrating the advantages in RNA side. These relatively modest AUROC values reflect the complexity of the protein–RNA prediction task due to the limited number of available protein–RNA complex structures. The difficulty stems from several critical factors. First, FoldBench consists of newly curated biological assemblies that were entirely unseen during the training phase of current models. Second, the dataset is subjected to a strict redundancy removal protocol, effectively eliminating evolutionary templates and forcing models to operate in the “twilight zone” where homology-based knowledge is unavailable. Furthermore, as noted in the FoldBench study, the redundancy removal is primarily centered on protein sequence identity, but less strict for RNA components. Thus, AF3 likely leverages a greater abundance of MSA and co-evolutionary signals for RNA binding nucleotide prediction. The results demonstrate ProRB’s ability to deliver the robust performance without 3D supervision or MSAs, highlighting its potential for analyzing data-sparse RBP targets where such co-evolutionary information is unavailable.

The advantage of ProRB’s sequence-based architecture is further evidenced by its computational efficiency (Fig. 3f). We compared the inference time of ProRB and AF3 across varying total sequence lengths (RNA + Protein). ProRB exhibits exceptional scalability, with inference times remaining consistently below 1 s ($ < {{10}^0}$ s), maintaining a several-order-of-magnitude speed advantage over AF3. The inference time for AF3 scales dramatically, ranging from hundreds to nearly ten thousand seconds (${{10}^2}$ – ${{10}^4}$) for larger complexes. This several-order-of-magnitude difference in speed underscores ProRB’s suitability for large-scale interactome screening and rapid *de novo* design. Regarding the computational memory usage, as AF3 was executed via the official web-based AlphaFold Server, direct hardware profiling of its peak GPU memory (VRAM) was unavailable. However, we evaluated the local memory of ProRB to showcase the inherent efficiency of our sequence-based model. Benefiting from a structure-free architecture, ProRB operates with an exceptionally low hardware barrier, requiring only a peak GPU memory (VRAM) of ~2.2 GB for a complex. This is much lower than the local deployment requirements of AF3, which typically demands GPUs with 40GB+ VRAM to handle large-scale spatial coordinate diffusion and MSA processing.

### ProRB captures motif-associated attention patterns for protein–RNA interactions

**Figure 4.**
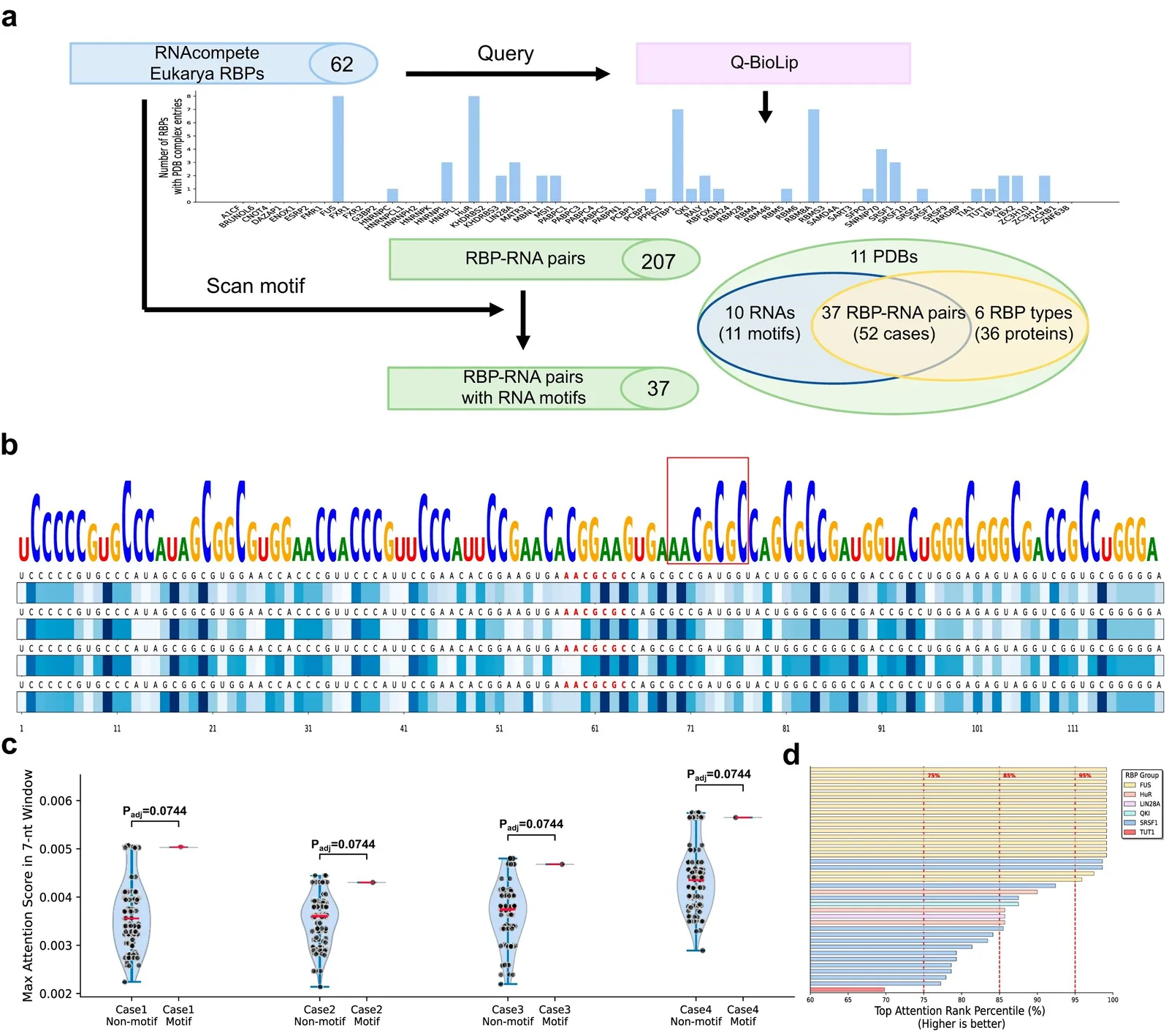
Cross-attention in ProRB reveals motif-centric recognition logic for protein–RNA interactions. (**a**) Analysis of 37 high-confidence protein–RNA complexes (11 families, 52 motif instances; FIMO *q* < 0.001, a false discovery rate-adjusted *P*-value threshold <.001), including 15 RNAs with 2 motifs. (**b**) For FUS (PDB: 4WQU), ProRB assigns high cross-attention scores to experimentally validated motif regions across four distinct protein–RNA pairs, visualized by nucleotide-size encoding (larger bases = higher attention). (**c**) Sliding-window analysis (7-nt window; sum of top-2 attention scores) shows motif regions exhibit higher attention scores versus non-motif regions across all four FUS cases (one-sided Wilcoxon, Benjamini–Hochberg adjusted *P*-value = .0744). (**d**) Dataset-wide motif saliency: the percentile rank of the highest attention score within each motif relative to its full RNA sequence (*n* = 37 complexes). For RNA sequences with two motifs, the peak attention scores are averaged. In 19/37 (51.4%), motif peaks reside in the top 5%; only one TUT1 complex falls outside the top 25%, but within the top 30%.

To evaluate whether ProRB’s attention distributions align with biologically documented RBP–RNA binding preferences, we conducted an interpretable analysis of its cross-attention patterns on a benchmark dataset derived directly from experimental sources. Specifically, we collected protein-binding motifs from the RNAcompete [23] compendium (62 RBPs with high-quality *in vitro* motifs) as queries to identify structurally resolved complexes in Q-BioLip [33]. This RBP-based matching yielded 11 proteins with available PDB structures, encompassing 37 distinct protein–RNA complexes. For each RNA, we scanned the corresponding RNAcompete-derived motif using FIMO [38] (*q* < 0.001), resulting in 52 high-confidence motif occurrences across the test set—including 15 RNAs with 2 motifs (Fig. 4a, Venn diagram).

We first examined the archetypal RBP FUS (PDB: 4WQU). Across four FUS–RNA complexes, which share identical or near-identical RNA sequences binding to different FUS protein variants, as detailed in Supplementary Table S11, ProRB yields consistent, sharp attention peaks over the motif regions in the protein (Fig. 4b). Visualized by nucleotide-sized glyphs (size ∝ attention score), motifs show marked enlargement relative to flanks, which are further reinforced by co-aligned attention heatmap (Fig. 4b, bottom). Moreover, although the same RNA sequence binds to four distinct and structurally divergent FUS proteins variants, the resulting RNA cross-attention heatmaps exhibit highly consistent peak locations despite variations in the protein context. While the high consistency of these RNA-side attention patterns is partially anticipated due to the near-identical RNA inputs, the stable alignment of attention peaks with the experimental motif across different protein variants indicates that ProRB robustly highlights these specific sequence regions during cross-modal integration.

To localize the motifs, we adopted a 7-nt sliding-window analysis, computing the top-2 attention score per window—a metric designed to capture the peak-centric nature of RBP binding. As shown in the side-by-side box plots (Fig. 4c), motif windows exhibit consistently higher peak attention than non-motif controls in all four FUS complexes (one-sided Wilcoxon, Benjamini–Hochberg adjusted *P*-value = .0744).

To assess the generalizability, we computed, for all 37 RBP–RNA complexes, the percentile rank of the highest attention score observed within each experimentally validated motif relative to the full RNA sequence (Fig. 4d). Notably, motif peaks rank in the top 5% for 19/37 complexes (51.4%), only one TUT1-associated complex falls outside the top 25%, yet remains within the top 30%. The results suggest that ProRB’s cross-attention layers preferentially target validated motif regions across diverse sequence contexts, supporting its utility in identifying biologically plausible binding sites. It is important to note that while these attention weights do not provide causal mechanistic explanations of physical binding, they demonstrate that ProRB learns robust statistical associations that align closely with known biological motifs.

### ProRB enables protein-binding RNA sequence design via conditional generation

Leveraging its motif-awareness, ProRB generates protein-conditioned RNA sequences via the decoder, originally trained with the teacher forcing during pretraining, which maps protein embeddings into an RNA latent space.

**Figure 5.**
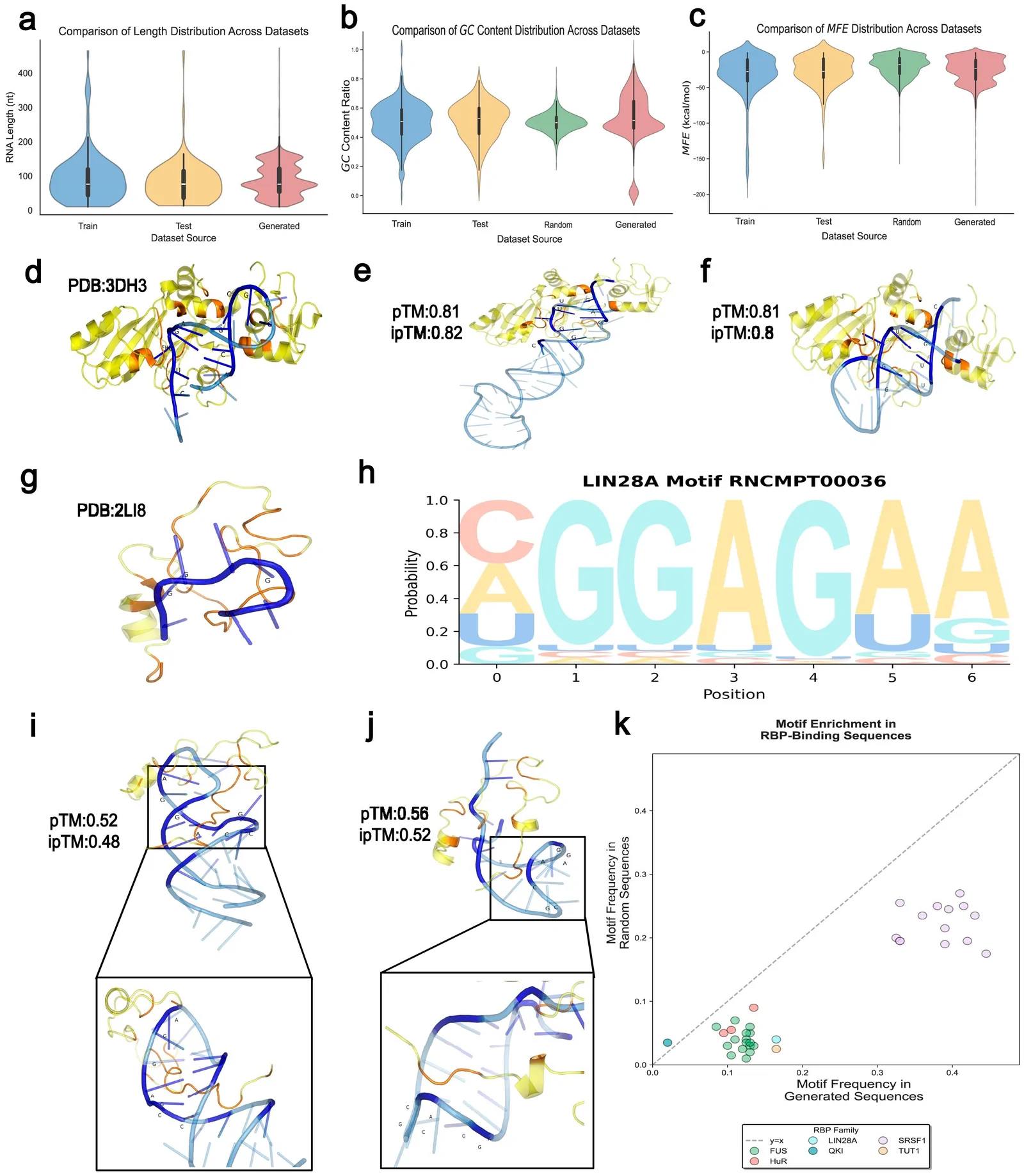
Visualization of ProRB-generated protein-binding RNA sequences and their predicted complexes by AF3. Comparison of biophysical properties among training, test, random, and ProRB-generated RNA sequences: (**a**) Sequence length distribution, (**b**) GC content, and (**c**) MFE for training RNA sequences, generated RNA sequences, and random RNA sequences. (**d**) Native RNA–protein complex (PDB: 3DH3). Predicted 3DH3-binding complexes for high-ranking ProRB designs: (**e**) Rank 1 candidate (pTM = 0.81, ipTM = 0.82) and (**f**) Rank 2 candidate (ipTM = 0.80). Both designs demonstrate exceptional structural fidelity and recover the essential base-pairing patterns at the interface. (**g**) Native LIN28A–RNA complex (PDB: 2LI8). (**h**) LIN28A motif (RNCMPT00036: 5′-GGAGAA-3′). Predicted LIN28A-binding complexes for high-ranking candidates in the data-scarce scenario: (i) Rank 1 design (pTM = 0.52, ipTM = 0.48) with the motif precisely positioned at the interface, and (**j**) Rank 2 design (pTM = 0.56, ipTM = 0.52) where the RNA backbone encapsulates the protein domain. (**k**) Systematic comparison of motif enrichment. Each point represents one of the 37 test RBP-RNA complexes. The *x*-axis denotes the percentage of ProRB-generated sequences (top 200 by PPL) containing the target RBP’s motif, while the *y*-axis shows the same for random controls.

To verify the distributional reasonableness of ProRB, we evaluated the statistical properties of the generated protein-binding RNA sequences against native (training/test) and random controls across three fundamental dimensions (Fig. 5a–c). As shown in Fig. 5a, the generated RNA sequences span a biophysical range and share a median length consistent with the native training and test datasets. However, unlike the smooth unimodal profile of the native datasets, the generated distribution exhibits a distinct multi-modal pattern characterized by multiple local peaks. This waving distribution is conceptually expected, since rather than collapsing to a generic, average-length template, the generator dynamically adapts its output to the sequence length scales preferred by different target proteins. After filtering out immediate-termination artifacts (*L = 0*, ${\mathrm{PPL}} = \infty $), the length distribution demonstrates that the active generator generally operates within the biologically plausible size range of native protein-binding RNAs rather than ignores the target protein’s conditional context and merely reproduces a blind statistical average. Quantitatively, the GC content of generated RNAs (53.05%) shows a slight upward shift compared to native levels (49.48% for training and 50.68% for test) and remains distinct from the random baseline (50.08%). To confirm that this GC enrichment is a learned feature of the ProRB rather than an artifact of sampling bias, we measured GC distributions across a wide temperature range (*T* = 0.4–1.6). As shown in Supplementary Fig. S4, the GC content remains consistently enriched even at higher temperatures. Regarding the thermodynamic stability (Fig. 5c), the mean MFE of the generated RNA sequences (−25.53 kcal/mol) closely mirrors the profile of the test set (−26.53 kcal/mol) and is markedly more stable than the random control (−20.51 kcal/mol). To decouple the influence of GC content from structural stability, we performed a GC-controlled comparison using dinucleotide-shuffled sequences. As illustrated in Supplementary Fig. S5, ProRB-generated sequences exhibit significantly lower MFE values compared to shuffled controls with identical GC content ($p = 1.93 \times {{10}^{ - 5}}$, Wilcoxon test), proving that the model captures higher-order structural logic beyond simple nucleotide composition.

To assess the structural plausibility of ProRB-generated RNA–protein complexes, we employed AF3 [24] to model the 3D structures of the designed RNAs with the targeted proteins. Prediction confidence was evaluated via predicted TM-score (pTM, global accuracy) and interface-predicted TM-score (ipTM, interface geometry).

For the case study of the RBP structure 3DH3 (Fig. 5d), we selected the top two generated RNA candidates according to the ranking score S_rank_ for explicit 3D structural validation using AF3 (Fig. 5e and f). Both high-ranking RNA sequences produced by ProRB demonstrate exceptional structural fidelity, the rank 1 sequence (Fig. 5e) achieves high confidence scores (pTM = 0.81; ipTM = 0.82), with its predicted binding geometry closely resembling the native complex. Similarly, the Rank 2 sequence (Fig. 5f) maintained a high ipTM of 0.80. Crucially, a detailed examination of the binding interface reveals that both generated RNA sequences successfully recover the essential secondary structural features of the native target. While the native RNA within the 3DH3 complex is characterized by specific base-pairing interactions at the interface (Fig. 5d), both ProRB-designed RNA candidates spontaneously formed equivalent base-pairing patterns in their predicted bound states. This structural alignment underscores the capacity of the ProRB framework to output highly plausible candidate sequences, demonstrating the utility of the multi-stage filtering protocol and integrated ranking strategy in computationally prioritizing promising designs. The multi-layered strategy ensures that the generated RNA candidates are not only compliant within the RNA sequence space but are also predicted to be compatible with the structural and biophysical constraints of the target protein interface. Crucially, we note that AF3 predictions serve as an assessment of structural plausibility rather than an absolute biological verification. Given that AF3 is itself a predictive model rather than experimental ground truth, the computational scores pTM and ipTM indicate geometric compatibility rather than definitive confirmation of actual binding activity or downstream biological function.

Moreover, to assess ProRB’s performance in data-scarce scenarios, we targeted LIN28A, an RBP for which only a single experimental complex (PDB: 2LI8, Fig. 5g) is currently available in the PDB, and the bound RNA comprises nearly the full motif (Fig. 5h). We selected two high-ranking candidates for structural validation via AF3 (Fig. 5i and j). The Rank 1 sequence (Fig. 5i) yielded pTM of 0.52 and ipTM of 0.48, where the generated binding motif is positioned precisely within the interface zone. Notably, this motif region undergoes spontaneous base-pairing with a distal segment of the RNA, mimicking the structural stabilization observed in native complexes. In the Rank 2 design (Fig. 5j, pTM = 0.56, ipTM = 0.52), the binding motif similarly occupies the core interface, with the RNA backbone forming a characteristic loop that encapsulates the protein domain to maximize sequence-specific contacts. The modestly lower confidence scores observed for LIN28A relative to 3DH3 are likely attributable to two factors: the extreme scarcity of structural templates, which limits the co-evolutionary signals available for AF3, and the high proportion of intrinsically disordered regions (IDRs) within the LIN28A protein. Such inherent flexibility often leads to the increased uncertainty in global orientation, resulting in lower pTM values. Nevertheless, the results show that ProRB consistently places the functional motifs at the interface and recovers biologically plausible secondary structures, suggesting that the model effectively internalizes the underlying recognition logic even for highly flexible and data-sparse targets.

To evaluate the generalizability of motif enrichment across diverse RBP families, we performed a systematic comparison between the top 200 ProRB-generated candidates prioritized by S_rank_ score and length-matched random controls for all 37 benchmark RBPs. As illustrated in Fig. 5k, the majority of RBP-conditioned designs cluster significantly below the diagonal, visually confirming that ProRB-generated RNAs are systematically enriched for functional motifs compared to random baselines. For instance, in the case of SRSF1, generated sequences achieved motif frequencies as high as 44.5%, markedly outperforming the random baseline of 17.5%. Similar trends were observed for LIN28A (16.5% versus 4.0%) and TUT1 (16.5% versus 2.5%), where functional motifs were enriched by approximately four- to six-fold. For the FUS and HuR families, although the absolute frequencies were more modest (averaging 10%–13%), they remained consistently higher than their respective random controls. We noted a specific exception for QKI, where the motif frequency was slightly lower than the random baseline (2.0% versus 3.5%). This suggests that for certain RBPs with highly specific or data-sparse binding grammars, the model may prioritize alternative structural features or non-canonical recognition patterns over the replication of simple linear motifs. Altogether, the enrichment of motifs across diverse RBP families confirms that the ProRB’s generative capability captures the essential sequence motifs required for specific protein–RNA recognition.

## Discussion

Understanding the binding properties of protein–RNA complexes is crucial for elucidating post-transcriptional regulation mechanisms and enabling the rational design of RNA therapeutics. In this study, we introduce ProRB, a unified sequence-based framework for protein–RNA interaction modeling, that jointly addresses the protein–RNA binding affinity prediction, binding site identification on proteins and RNAs, and motif-preserving *de novo* protein-binding RNA sequences design. ProRB achieves state-of-the-art performance on the binding affinity prediction, outperforming structure-based baselines like CoPRA and the recently published large-scale multi-omic model OmniBioTE. For dual-modal binding site prediction, ProRB not only surpasses structure-based methods like Frame Averaging across CD-HIT clustering thresholds from 40% to 90%, but also demonstrates the interface recognition performance competitive with AF3 on the stringent FoldBench dataset. While ProRB shows a slight advantage in protein-side residue prediction and offers a several-order-of-magnitude speedup, its performance on the RNA-side interface remains lower than that of AF3. One potential reason is that structure-based approaches like AF3 can leverage multimodal input like MSA and well-designed modules like Invariant Point Attention, which are critical for capturing the intricate, non-linear structural motifs of RNA interfaces. Conversely, sequence-based models like ProRB bypass the need for MSA templates and are highly scalable, but they are intrinsically limited in capturing conformational changes, induced-fit thermodynamics, and spatial-geometry details that are not fully encoded in primary sequences. Furthermore, we explicitly acknowledge that the FoldBench comparison is based on a small dataset size, where the comparative results should be viewed as illustrative rather than definitive. For RNA design, ProRB generates protein-binding RNA sequences with enhanced biophysical properties (e.g. higher GC content and more stable MFE) and motif enrichment. The results further indicate that the synergy between ProRB’s generative capacity and the multi-stage filtering-ranking protocol is vital for enriching motif-preserving RNA candidates, effectively narrowing down the candidate space to filter out biophysical noise prior to experimental validation.

Our analysis highlights how foundation model priors and designed information interaction mechanism effectively compensate with absent 3D structure data in protein–RNA binding affinity and interfaces prediction, and protein-binding RNA sequence design. The success of this unified framework is rooted in its hierarchical feature organization, which balances shared foundational knowledge with task-specific logic. Specifically, all three tasks share a core representation space, utilizing high-dimensional evolutionary priors from ESMC and RNA-FM as well as a protein-conditioned RNA latent representation ${{E}_{\mathrm{fakeRNA}}}$ that serves as a shared interaction context. Unifying the three tasks into a single framework proves highly beneficial, as demonstrated by the improved protein–RNA interaction predictions and motif-centric interpretability. While the foundation is shared, ProRB processes these features at distinct granularities: it captures the comprehensive “energetic logic” of binding affinity via a global interaction matrix through outer product, while identifying binding sites through local, dual-modal refined embeddings updated via cross-attention mechanism. Regarding the potential data overlap between our evaluation datasets and the pretraining corpora of the baseline foundation models, we explicitly acknowledge that since ESMC and RNA-FM are respectively pretrained on billions of evolutionary sequences and the entire RNAcentral database, the primary sequences of structurally resolved complexes from the PDB are inevitably included within their massive pretraining process. Therefore, sequence-level overlap inherently exists. To mitigate concerns that this overlap might artificially inflate the interpretation of the generalization performance, we evaluated the framework under strict sequence redundancy, implementing strict sequence identity cutoffs (20% and 25%) and structural fold-similarity thresholds (40% and 50%) for affinity prediction, alongside stringent sequence de-redundancy (40%) for dual-modal binding sites prediction. Intriguingly, the omission of either the cross-attention or self-attention modules results in a more severe performance collapse in both prediction tasks, revealing that the relational interaction model architecture, rather than the raw foundation model priors, plays the dominant role in model generalization. For the generative task, the model specifically utilizes the autoregressive feedback loop within the decoder, treating ${{E}_{\mathrm{fakeRNA}}}$ as a functional template to guide RNA sequence design. This integration helps ProRB outperform isolated approaches by capturing relational priors, yielding more robust generalization and interpretable insights.

Nevertheless, key challenges persist, particularly regarding dataset constraints. First, the current binding affinity benchmark PRA201 remains relatively small, comprising only 201 protein–RNA complexes, which inherently limits the upper bound of statistical confidence. Moreover, although ProRB achieves the state-of-the-art performance among baselines, we explicitly clarify that our performance claims are strictly restricted to the subset of methods that could be successfully reproduced and benchmarked under the current evaluation framework. Furthermore, the observed Pearson correlation of ∼0.53 across de-redundancy thresholds implies that a substantial fraction of binding variance remains unexplained. Second, sequence-based models capture recognition logic well but struggle with dynamics like induced-fit or allostery, which require structural plasticity beyond primary sequences. Third, capturing recognition logic in data-sparse or structurally complicate families remains a challenge. While the systematic motif enrichment analysis across 37 RBPs confirms that ProRB consistently outperforms random controls for the families like FUS and SRSF1, it also reveals inferior performance for proteins such as QKI and TUT1. This discrepancy highlights that for certain RBPs, sequence-only priors may be insufficient to fully decouple functional motifs from background noise, especially when the binding is driven by rare structural conformations. Finally, a notable constraint of the current ProRB framework lies in the sequence length thresholds applied to the PRI30K pretraining dataset, the dataset is restricted to proteins ≤750 amino acids and RNAs between 5 and 500 nucleotides. While these constraints are computationally necessary to ensure attention map scalability and prevent training instability, they inevitably restrict the model’s immediate applicability to longer, biologically pivotal macromolecules. Specifically, many key RBPs, such as large helicases or multi-domain splicing regulators, exceed 750 amino acids, and major classes of non-coding transcripts, such as long non-coding RNAs, routinely span thousands of nucleotides. Although ProRB’s sequence-only, structure-free architecture bypasses the stringent coordinate-matching requirements of 3D-based models, extreme length scales introduce dual bottlenecks: quadratic memory scaling inherent to standard Transformer attention, and potential representation dilution over long-range dependencies where sparse functional motifs are embedded within vast uninformative sequences. Consequently, the zero-shot generalizability of the current checkpoint to macromolecular complexes characterized by exceptionally long proteins or RNA chains remains constrained. Future work will focus on mitigating these limitations by integrating memory-efficient linear or sliding-window attention mechanisms, and employing chunk-based or hierarchical encoding strategies, thereby extending ProRB’s decoding capabilities to the scale of long non-coding RNA-RBP interactomes.

Moreover, the metrics for evaluating the quality of generated protein-binding RNA sequences are currently insufficient for comprehensive functional assessment, as properties like length, GC content, and MFE only provide statistical context regarding the sequence fluency rather than binding plausibility. In respect of structural plausibility, we explicitly acknowledge the methodological limitation and potential circularity in relying on AF3 for wet-lab validation. As a deep-learning-based structure predictor, AF3 provides invaluable insights into spatial reasonability at the interface, but it cannot substitute for experimental ground truth. Reliance on pTM and ipTM metrics across case studies may overestimate binding outcomes, as these predictive metrics do not capture true binding kinetics, induced-fit thermodynamics, or specific biological functionality. In conclusion, while ProRB utilizes a dynamic length-control mechanism to learn these computational patterns implicitly and the designed protein-binding RNA sequences display high sequence plausibility, foldability, and predicted interaction compatibility, the sequences generated by ProRB should be treated as computationally prioritized RNA candidates rather than experimentally validated binders, their biological function and binding specificity require further empirical confirmation and subsequent experimental validation via molecular dynamics simulations and wet-lab techniques remain essential to confirm the kinetic stability and functions of the designed RNAs.

Furthermore, the role of sequence mutations can serve as a critical bridge between theoretical design and functional optimization. In the ProRB framework, mutation sensitivity is intrinsically captured through the adaptive cross-modal attention mechanism, where nucleotides with high attention scores are critical interaction hubs. This enables a mutation-guided design strategy: by identifying these sensitive locations, researchers can prioritize the preservation of essential functional motifs while selectively mutating non-critical flanking regions to improve biophysical properties. Rather than relying on trial-and-error, ProRB navigates the mutational landscape by filtering out substitutions that disrupt the learned biophysical grammar, as reflected by high perplexity scores.

In summary, ProRB advances scalable protein–RNA interaction modeling. Although protein–RNA affinity and interface prediction remain challenging, the reported performance gains of ProRB hold profound practical biological significance, particularly for virtual screening and rational protein-binding RNA design. In practical settings, the primary bottleneck in designing small activating RNAs or RBP-targeted aptamers is the prohibitive cost of wet-lab trial-and-error, where ProRB can serve as a highly efficient computational filter. It empowers biophysicists to robustly prioritize and rank the interaction potential of thousands of candidate RNA sequences conditioned on a target protein sequence without requiring 3D structural modeling and significantly accelerates the optimization loop for motif-guided RNA therapeutics by predicting potential binding sites. Future efforts include structure-conditioned fine-tuning of the decoder with constraints (e.g. distance/torsion from cryo-EM or SHAPE probing), yielding structurally precise RNA sequence designs. This integration will link motif recognition to systems regulation, enabling rational design of RNA therapeutics for dysregulated RBP pathways.

## Supplementary Material

gkag870_Supplemental_File

## Data Availability

PRI30K and PRA201 datasets were from https://huggingface.co/datasets/Jesse7/CoPRA_data/tree/main. Binding sites-related datasets were from https://yanglab.qd.sdu.edu.cn/Q-BioLiP/. FoldBench dataset was from https://github.com/BEAM-Labs/FoldBench. ProRB is open-source and available at https://doi.org/10.5281/zenodo.21447682.
