# Supplementary material for: ProRB: a structure-free unified framework for joint prediction and design of protein–RNA interactions
Source: Nucleic Acids Res. 2026 Sep 7;54(17):gkag870. doi: 10.1093/nar/gkag870 (PMC13548063; doi:10.1093/nar/gkag870)
Supplement: Supplementary file 1 [file gkag870_supplemental_file.docx]

# Supplementary material

***Supplementary Figures***

Figure S1. Statistical distributions of unique clusters, training samples, and test samples across all identity thresholds.

Figure S2. Detailed workflow of the training and inference phases for protein-binding RNA sequence generation.

Figure S3. Statistical significance for comparisons of key protein-RNA binding affinity prediction baselines.

Figure S4. GC content across different sampling temperatures.

Figure S5. Comparison of MFE distributions between ProRB-generated and GC-matched shuffled RNA sequences.

***Supplementary Tables***

Table S1 Protein vocabulary

| token | id |
| --- | --- |
| <mask> | 32 |
| \| | 31 |
| - | 30 |
| . | 29 |
| O | 28 |
| Z | 27 |
| U | 26 |
| B | 25 |
| X | 24 |
| C | 23 |
| W | 22 |
| H | 21 |
| M | 20 |
| Y | 19 |
| F | 18 |
| N | 17 |
| Q | 16 |
| K | 15 |
| P | 14 |
| D | 13 |
| I | 12 |
| T | 11 |
| R | 10 |
| E | 9 |
| S | 8 |
| V | 7 |
| G | 6 |
| A | 5 |
| L | 4 |
| <unk> | 3 |
| <eos> | 2 |
| <pad> | 1 |
| <cls> | 0 |

Table S2 RNA vocabulary

| token | id |
| --- | --- |
| I | 25 |
| - | 24 |
| * | 23 |
| X | 22 |
| . | 21 |
| V | 20 |
| H | 19 |
| D | 18 |
| B | 17 |
| M | 16 |
| K | 15 |
| W | 14 |
| S | 13 |
| Y | 12 |
| R | 11 |
| N | 10 |
| U | 9 |
| G | 8 |
| C | 7 |
| A | 6 |
| <null> | 5 |
| <mask> | 4 |
| <unk> | 3 |
| <eos> | 2 |
| <cls> | 1 |
| <pad> | 0 |

Table S3 Performance of all baselines in predicting binding affinity.

| threshold_type | threshold | method | mae | rmse | pearson | spearman |
| --- | --- | --- | --- | --- | --- | --- |
| Sequence | 20 | CoPRA | 1.4087 ± 0.2455 | 1.7614 ± 0.2546 | 0.2866 ± 0.1741 | 0.3163 ± 0.1761 |
| Sequence | 20 | DeepNAP | 2.1784 ± 1.2933 | 2.3472 ± 1.1970 | 0.0907 ± 0.1032 | 0.0544 ± 0.0975 |
| Sequence | 20 | OmniBioTE | 1.1907 ± 0.1615 | 1.4556 ± 0.1998 | 0.5061 ± 0.0970 | 0.5356 ± 0.0991 |
| Sequence | 20 | ProRB | 1.1467 ± 0.2816 | 1.3766 ± 0.3649 | 0.5347 ± 0.0741 | 0.5317 ± 0.0773 |
| Sequence | 25 | CoPRA | 1.3054 ± 0.1111 | 1.6215 ± 0.1105 | 0.3535 ± 0.0911 | 0.4027 ± 0.1012 |
| Sequence | 25 | DeepNAP | 2.1182 ± 1.1236 | 2.2560 ± 1.0570 | 0.1072 ± 0.1094 | 0.0799 ± 0.1300 |
| Sequence | 25 | OmniBioTE | 1.1866 ± 0.1603 | 1.5419 ± 0.1966 | 0.4297 ± 0.1202 | 0.4471 ± 0.1800 |
| Sequence | 25 | ProRB | 1.1415 ± 0.2875 | 1.4021 ± 0.3541 | 0.5274 ± 0.0697 | 0.5198 ± 0.0678 |
| Structure | 40 | CoPRA | 1.3015 ± 0.1165 | 1.6187 ± 0.1138 | 0.3586 ± 0.0871 | 0.4044 ± 0.0994 |
| Structure | 40 | DeepNAP | 1.5623 ± 0.3930 | 1.7854 ± 0.4920 | 0.0960 ± 0.1343 | 0.0560 ± 0.1151 |
| Structure | 40 | OmniBioTE | 1.3973 ± 0.1335 | 1.7171 ± 0.1219 | 0.1726 ± 0.1603 | 0.1349 ± 0.2243 |
| Structure | 40 | ProRB | 1.2048 ± 0.2056 | 1.3759 ± 0.3242 | 0.5060 ± 0.1138 | 0.4821 ± 0.1068 |
| Structure | 50 | CoPRA | 1.2902 ± 0.1341 | 1.6067 ± 0.1307 | 0.3702 ± 0.0834 | 0.4198 ± 0.0884 |
| Structure | 50 | DeepNAP | 2.2065 ± 0.9168 | 2.4600 ± 0.9115 | -0.0117 ± 0.2357 | 0.0036 ± 0.2065 |
| Structure | 50 | OmniBioTE | 1.2843 ± 0.1975 | 1.5878 ± 0.2193 | 0.3834 ± 0.1928 | 0.3836 ± 0.1176 |
| Structure | 50 | ProRB | 1.2394 ± 0.2714 | 1.3825 ± 0.4102 | 0.4643 ± 0.1331 | 0.4613 ± 0.1430 |

Table S4 Performance of models w/ and w/o pretraining in predicting binding affinity.

| threshold | method | mae | rmse | pearson | spearman |
| --- | --- | --- | --- | --- | --- |
| 20 | ProRB | 1.1467 ± 0.2816 | 1.3766 ± 0.3649 | 0.5347 ± 0.0741 | 0.5317 ± 0.0773 |
| 20 | w/o pretrain | 1.2323 ± 0.2227 | 1.4588 ± 0.1973 | 0.3785 ± 0.1166 | 0.3751 ± 0.1422 |
| 25 | ProRB | 1.1415 ± 0.2875 | 1.4021 ± 0.3541 | 0.5274 ± 0.0697 | 0.5198 ± 0.0678 |
| 25 | w/o pretrain | 1.2225 ± 0.2392 | 1.4558 ± 0.2001 | 0.3754 ± 0.1159 | 0.3706 ± 0.1395 |
| 40 | ProRB | 1.2048 ± 0.2056 | 1.3759 ± 0.3242 | 0.5060 ± 0.1138 | 0.4821 ± 0.1068 |
| 40 | w/o pretrain | 1.3551 ± 0.5020 | 1.3772 ± 0.5167 | 0.3928 ± 0.0531 | 0.3873 ± 0.1068 |
| 50 | ProRB | 1.2394 ± 0.2714 | 1.3825 ± 0.4102 | 0.4643 ± 0.1331 | 0.4613 ± 0.1430 |
| 50 | w/o pretrain | 1.4383 ± 0.5273 | 1.5880 ± 0.6586 | 0.3243 ± 0.1118 | 0.3051 ± 0.1257 |

Table S5 Performance of ablations in predicting binding affinity.

| Redundancy_Type | Threshold | Method | MAE | RMSE | Pearson | Spearman |
| --- | --- | --- | --- | --- | --- | --- |
| Sequence | 20 | Full Model | 1.1467 ± 0.2816 | 1.3766 ± 0.3649 | 0.5347 ± 0.0741 | 0.5317 ± 0.0773 |
| Sequence | 20 | w/o Cross-Attention | 1.3121 ± 0.6470 | 1.3460 ± 0.6409 | 0.3438 ± 0.0943 | 0.3394 ± 0.1490 |
| Sequence | 20 | w/o Self-Attention | 1.3432 ± 0.3568 | 1.3737 ± 0.3626 | 0.3299 ± 0.1797 | 0.3463 ± 0.1748 |
| Sequence | 20 | One-Hot Encoding | 1.2845 ± 0.2621 | 1.3917 ± 0.3236 | 0.3854 ± 0.1346 | 0.3991 ± 0.1154 |
| Sequence | 25 | Full Model | 1.1415 ± 0.2875 | 1.4021 ± 0.3541 | 0.5274 ± 0.0697 | 0.5198 ± 0.0678 |
| Sequence | 25 | w/o Cross-Attention | 1.3493 ± 0.1940 | 1.3934 ± 0.1807 | 0.3342 ± 0.1124 | 0.3383 ± 0.1195 |
| Sequence | 25 | w/o Self-Attention | 1.3774 ± 0.3938 | 1.4445 ± 0.4258 | 0.3754 ± 0.1259 | 0.3561 ± 0.1603 |
| Sequence | 25 | One-Hot Encoding | 1.1115 ± 0.2645 | 1.4237 ± 0.4963 | 0.3827 ± 0.1363 | 0.3983 ± 0.1159 |
| Structural | 40 | Full Model | 1.2048 ± 0.2056 | 1.3759 ± 0.3242 | 0.5060 ± 0.1138 | 0.4821 ± 0.1068 |
| Structural | 40 | w/o Cross-Attention | 1.3786 ± 0.3183 | 1.4539 ± 0.3660 | 0.3819 ± 0.0639 | 0.3651 ± 0.0893 |
| Structural | 40 | w/o Self-Attention | 1.3798 ± 0.4060 | 1.4081 ± 0.4299 | 0.3934 ± 0.0587 | 0.3793 ± 0.0790 |
| Structural | 40 | One-Hot Encoding | 1.2688 ± 0.4137 | 1.4527 ± 0.4800 | 0.3873 ± 0.1050 | 0.3873 ± 0.1517 |
| Structural | 50 | Full Model | 1.2394 ± 0.2714 | 1.3825 ± 0.4102 | 0.4643 ± 0.1331 | 0.4613 ± 0.1430 |
| Structural | 50 | w/o Cross-Attention | 1.4061 ± 0.5986 | 1.5398 ± 0.7156 | 0.3454 ± 0.0521 | 0.3289 ± 0.0392 |
| Structural | 50 | w/o Self-Attention | 1.4105 ± 0.6027 | 1.5276 ± 0.7353 | 0.2850 ± 0.1139 | 0.2821 ± 0.0913 |
| Structural | 50 | One-Hot Encoding | 1.4064 ± 0.5786 | 1.6241 ± 0.7347 | 0.3271 ± 0.1686 | 0.4205 ± 0.1811 |

Table S6 Performance of ProRB’s different alpha in predicting binding affinity.

| Threshold | Alpha | MAE | RMSE | Pearson | Spearman |
| --- | --- | --- | --- | --- | --- |
| 20 | 0 | 1.3210 ± 0.4442 | 1.5782 ± 0.4569 | 0.4333 ± 0.1629 | 0.4257 ± 0.1776 |
| 20 | 0.2 | 1.3730 ± 0.3669 | 1.6764 ± 0.3986 | 0.3614 ± 0.1246 | 0.3225 ± 0.1395 |
| 20 | 0.4 | 1.3960 ± 0.2403 | 1.6924 ± 0.2724 | 0.4369 ± 0.1328 | 0.4277 ± 0.1378 |
| 20 | 0.6 | 1.4345 ± 0.2702 | 1.6169 ± 0.3233 | 0.4358 ± 0.1000 | 0.4427 ± 0.1145 |
| 20 | 0.8 | 1.1467 ± 0.2816 | 1.3766 ± 0.3649 | 0.5347 ± 0.0741 | 0.5317 ± 0.0773 |
| 20 | 1 | 1.2939 ± 0.4507 | 1.6007 ± 0.5559 | 0.3759 ± 0.2040 | 0.3668 ± 0.1968 |
| 25 | 0 | 1.1192 ± 0.3258 | 1.3844 ± 0.3734 | 0.4097 ± 0.1778 | 0.3886 ± 0.2048 |
| 25 | 0.2 | 1.2777 ± 0.3534 | 1.5316 ± 0.4349 | 0.3885 ± 0.1442 | 0.3705 ± 0.1537 |
| 25 | 0.4 | 1.3437 ± 0.2032 | 1.6168 ± 0.2484 | 0.4435 ± 0.1477 | 0.4409 ± 0.1649 |
| 25 | 0.6 | 1.2858 ± 0.1849 | 1.4986 ± 0.2098 | 0.4668 ± 0.0618 | 0.4665 ± 0.1029 |
| 25 | 0.8 | 1.1415 ± 0.2875 | 1.4021 ± 0.3541 | 0.5274 ± 0.0697 | 0.5198 ± 0.0678 |
| 25 | 1 | 1.3171 ± 0.4439 | 1.6137 ± 0.5372 | 0.3861 ± 0.2059 | 0.3774 ± 0.1900 |
| 40 | 0 | 1.4096 ± 0.4341 | 1.5488 ± 0.3964 | 0.4619 ± 0.0666 | 0.4388 ± 0.0975 |
| 40 | 0.2 | 1.5002 ± 0.2966 | 1.6236 ± 0.3159 | 0.4191 ± 0.1008 | 0.3983 ± 0.0905 |
| 40 | 0.4 | 1.2016 ± 0.2618 | 1.3268 ± 0.2582 | 0.3521 ± 0.0886 | 0.2648 ± 0.1935 |
| 40 | 0.6 | 1.4492 ± 0.6195 | 1.5456 ± 0.5776 | 0.3811 ± 0.0998 | 0.4113 ± 0.1210 |
| 40 | 0.8 | 1.2048 ± 0.2056 | 1.3759 ± 0.3242 | 0.5060 ± 0.1138 | 0.4821 ± 0.1068 |
| 40 | 1 | 1.2673 ± 0.3239 | 1.3784 ± 0.3360 | 0.3807 ± 0.1133 | 0.3654 ± 0.1236 |
| 50 | 0 | 1.2524 ± 0.3950 | 1.4729 ± 0.4232 | 0.4186 ± 0.1417 | 0.4069 ± 0.1508 |
| 50 | 0.2 | 1.3836 ± 0.2807 | 1.5973 ± 0.3963 | 0.4091 ± 0.1066 | 0.3747 ± 0.0999 |
| 50 | 0.4 | 1.3183 ± 0.3362 | 1.4743 ± 0.4051 | 0.4162 ± 0.0842 | 0.4163 ± 0.0926 |
| 50 | 0.6 | 1.3853 ± 0.3137 | 1.5707 ± 0.4628 | 0.3667 ± 0.1470 | 0.3578 ± 0.1595 |
| 50 | 0.8 | 1.2394 ± 0.2714 | 1.3825 ± 0.4102 | 0.4643 ± 0.1331 | 0.4613 ± 0.1430 |
| 50 | 1 | 1.4096 ± 0.2915 | 1.5481 ± 0.3761 | 0.4208 ± 0.0778 | 0.4069 ± 0.1057 |

Table S7 Performance of ProRB and Frame Averaging in predicting binding interface.

| Threshold | Method | | RNA_AUC  (mean/std) | prot_AUC  (mean/std) | RNA_AUPRC  (mean/std) | prot_AUPRC  (mean/std) |
| --- | --- | --- | --- | --- | --- | --- |
| 40 | FA | 0.7517 / 0.0204 | | 0.6194 / 0.0356 | 0.2560 / 0.0134 | 0.0807 / 0.0065 |
| 40 | ProRB | 0.7565 / 0.0166 | | 0.6841 / 0.0140 | 0.2656 / 0.0117 | 0.1220 / 0.0127 |
| 50 | FA | 0.6958 / 0.0274 | | 0.6219 / 0.0331 | 0.1984 / 0.0208 | 0.0966 / 0.0060 |
| 50 | ProRB | 0.7174 / 0.0180 | | 0.6926 / 0.0154 | 0.2077 / 0.0085 | 0.1305 / 0.0221 |
| 60 | FA | 0.7045 / 0.0357 | | 0.6313 / 0.0584 | 0.2124 / 0.0434 | 0.0989 / 0.0205 |
| 60 | ProRB | 0.7176 / 0.0237 | | 0.7030 / 0.0229 | 0.2331 / 0.0380 | 0.1394 / 0.0240 |

Table S8 Performance of ablations in predicting binding interface.

| Threshold | | Method | RNA_AUC  (mean/std) | | prot_AUC  (mean/std) | | RNA_AUPRC  (mean/std) | | prot_AUPRC  (mean/std) | |  |
| --- | --- | --- | --- | --- | --- | --- | --- | --- | --- | --- | --- |
| 40 | One-hot | | | 0.6624/0.0224 | | 0.6822/0.0081 | | 0.1517/0.0180 | | 0.1288/0.0201 | |
| 40 | w/o cross_attn | | | 0.7490/0.0275 | | 0.5301/0.0290 | | 0.2466/0.0185 | | 0.0572/0.0052 | |
| 40 | w/o self_attn | | | 0.7490/0.0276 | | 0.5398/0.0453 | | 0.2471/0.0185 | | 0.0617/0.0107 | |
| 40 | ProRB | | | 0.7565/0.0166 | | 0.6841/0.0140 | | 0.2656/0.0117 | | 0.1220/0.0127 | |
| 50 | One-hot | | | 0.6079/0.0329 | | 0.6868/0.0107 | | 0.1341/0.0170 | | 0.1380/0.0199 | |
| 50 | w/o cross_attn | | | 0.6964/0.0200 | | 0.5374/0.0202 | | 0.1962/0.0133 | | 0.0667/0.0055 | |
| 50 | w/o self_attn | | | 0.6964/0.0200 | | 0.5373/0.0204 | | 0.1962/0.0134 | | 0.0667/0.0054 | |
| 50 | ProRB | | | 0.7174/0.0180 | | 0.6926/0.0154 | | 0.2077/0.0085 | | 0.1305/0.0221 | |
| 60 | One-hot | | | 0.5995/0.0403 | | 0.6830/0.0164 | | 0.1313/0.0172 | | 0.1341/0.0194 | |
| 60 | w/o cross_attn | | | 0.7041/0.0378 | | 0.5281/0.0358 | | 0.2094/0.0522 | | 0.0643/0.0115 | |
| 60 | w/o self_attn | | | 0.7042/0.0379 | | 0.5282/0.0358 | | 0.2095/0.0523 | | 0.0643/0.0115 | |
| 60 | ProRB | | | 0.7176/0.0237 | | 0.7030/0.0229 | | 0.2331/0.0380 | | 0.1394/0.0240 | |
| 70 | One-hot | | | 0.6024/0.0107 | | 0.7009/0.0112 | | 0.1203/0.0095 | | 0.1559/0.0263 | |
| 70 | w/o cross_attn | | | 0.6939/0.0193 | | 0.5403/0.0179 | | 0.1927/0.0212 | | 0.0664/0.0031 | |
| 70 | w/o self_attn | | | 0.6939/0.0194 | | 0.5403/0.0178 | | 0.1927/0.0212 | | 0.0663/0.0031 | |
| 70 | ProRB | | | 0.7177/0.0171 | | 0.7130/0.0185 | | 0.2119/0.0200 | | 0.1604/0.0218 | |
| 80 | One-hot | | | 0.5986/0.0282 | | 0.6880/0.0157 | | 0.1286/0.0131 | | 0.1432/0.0173 | |
| 80 | w/o cross_attn | | | 0.7125/0.0147 | | 0.5776/0.0270 | | 0.2093/0.0101 | | 0.0758/0.0057 | |
| 80 | w/o self_attn | | | 0.7121/0.0151 | | 0.5702/0.0259 | | 0.2098/0.0097 | | 0.0742/0.0083 | |
| 80 | ProRB | | | 0.7318/0.0124 | | 0.7192/0.0200 | | 0.2344/0.0127 | | 0.1614/0.0213 | |
| 90 | One-hot | | | 0.6405/0.0379 | | 0.6923/0.0084 | | 0.1573/0.0293 | | 0.1556/0.0080 | |
| 90 | w/o cross_attn | | | 0.7293/0.0209 | | 0.5861/0.0618 | | 0.2254/0.0247 | | 0.0839/0.0191 | |
| 90 | w/o self_attn | | | 0.7292/0.0208 | | 0.5861/0.0619 | | 0.2249/0.0265 | | 0.0841/0.0195 | |
| 90 | ProRB | | | 0.7529/0.0189 | | 0.7205/0.0191 | | 0.2512/0.0294 | | 0.1706/0.0126 | |

Table S9 Performance of models w/ and w/o pretraining in predicting binding interface.

| Threshold | Method | RNA_AUC  (mean/std) | prot_AUC  (mean/std) | RNA_AUPRC  (mean/std) | prot_AUPRC  (mean/std) |
| --- | --- | --- | --- | --- | --- |
| 40 | w/o pretraining | 0.7473/0.0218 | 0.6029/0.0323 | 0.2442/0.0229 | 0.0796/0.0090 |
| 40 | w/ pretraining | 0.7565/0.0166 | 0.6841/0.0140 | 0.2656/0.0117 | 0.1220/0.0127 |
| 50 | w/o pretraining | 0.6959/0.0234 | 0.6017/0.0259 | 0.1937/0.0177 | 0.0955/0.0141 |
| 50 | w/ pretraining | 0.7174/0.0180 | 0.6926/0.0154 | 0.2077/0.0085 | 0.1305/0.0221 |
| 60 | w/o pretraining | 0.7022/0.0337 | 0.6258/0.0557 | 0.2051/0.0399 | 0.0955/0.0189 |
| 60 | w/ pretraining | 0.7176/0.0237 | 0.7030/0.0229 | 0.2331/0.0380 | 0.1394/0.0240 |
| 70 | w/o pretraining | 0.7018/0.0236 | 0.6829/0.0324 | 0.1950/0.0200 | 0.1258/0.0218 |
| 70 | w/ pretraining | 0.7177/0.0171 | 0.7130/0.0185 | 0.2119/0.0200 | 0.1604/0.0218 |
| 80 | w/o pretraining | 0.7249/0.0114 | 0.6925/0.0337 | 0.2177/0.0074 | 0.1327/0.0287 |
| 80 | w/ pretraining | 0.7318/0.0124 | 0.7192/0.0200 | 0.2344/0.0127 | 0.1614/0.0213 |
| 90 | w/o pretraining | 0.7441/0.0170 | 0.6973/0.0164 | 0.2402/0.0258 | 0.1383/0.0151 |
| 90 | w/ pretraining | 0.7529/0.0189 | 0.7205/0.0191 | 0.2512/0.0294 | 0.1706/0.0126 |

Table S10 Performance of ProRB’s different alpha in predicting binding interface.

| Threshold | | Alpha | RNA_AUC  (mean/std) | | prot_AUC  (mean/std) | | | RNA_AUPRC  (mean/std) | | prot_AUPRC  (mean/std) | |
| --- | --- | --- | --- | --- | --- | --- | --- | --- | --- | --- | --- |
| 40 | 0 | | | 0.7539/0.0171 | | 0.6843/0.0229 | 0.2452/0.0187 | | 0.1120/0.0052 | |  |
| 40 | 0.2 | | | 0.7516/0.0177 | | 0.6941/0.0202 | 0.2387/0.0164 | | 0.1240/0.0093 | |  |
| 40 | 0.4 | | | 0.7486/0.0217 | | 0.6874/0.0261 | 0.2481/0.0184 | | 0.1198/0.0067 | |  |
| 40 | 0.6 | | | 0.7544/0.0157 | | 0.6890/0.0217 | 0.2440/0.0194 | | 0.1177/0.0157 | |  |
| 40 | 0.8 | | | 0.7565/0.0166 | | 0.6841/0.0140 | 0.2656/0.0117 | | 0.1220/0.0127 | |  |
| 40 | 1 | | | 0.7494/0.0131 | | 0.6910/0.0280 | 0.2388/0.0249 | | 0.1179/0.0130 | |  |
| 50 | 0 | | | 0.7188/0.0238 | | 0.6958/0.0172 | 0.2040/0.0240 | | 0.1296/0.0291 | |  |
| 50 | 0.2 | | | 0.7103/0.0264 | | 0.7062/0.0091 | 0.1965/0.0196 | | 0.1339/0.0157 | |  |
| 50 | 0.4 | | | 0.7094/0.0274 | | 0.6874/0.0185 | 0.1923/0.0242 | | 0.1179/0.0167 | |  |
| 50 | 0.6 | | | 0.7156/0.0288 | | 0.6902/0.0117 | 0.2050/0.0246 | | 0.1272/0.0197 | |  |
| 50 | 0.8 | | | 0.7174/0.0180 | | 0.6926/0.0154 | 0.2077/0.0085 | | 0.1305/0.0221 | |  |
| 50 | 1 | | | 0.7108/0.0194 | | 0.7018/0.0226 | 0.1970/0.0179 | | 0.1417/0.0256 | |  |
| 60 | 0 | | | 0.7195/0.0249 | | 0.6990/0.0270 | 0.2296/0.0411 | | 0.1267/0.0188 | |  |
| 60 | 0.2 | | | 0.7113/0.0222 | | 0.7089/0.0284 | 0.2228/0.0358 | | 0.1334/0.0180 | |  |
| 60 | 0.4 | | | 0.7144/0.0243 | | 0.6969/0.0270 | 0.2201/0.0363 | | 0.1270/0.0200 | |  |
| 60 | 0.6 | | | 0.7164/0.0363 | | 0.6975/0.0317 | 0.2298/0.0392 | | 0.1255/0.0195 | |  |
| 60 | 0.8 | | | 0.7176/0.0237 | | 0.7030/0.0229 | 0.2331/0.0380 | | 0.1394/0.0240 | |  |
| 60 | 1 | | | 0.7089/0.0347 | | 0.7056/0.0307 | 0.2211/0.0475 | | 0.1392/0.0232 | |  |
| 70 | 0 | | | 0.7155/0.0166 | | 0.7236/0.0213 | 0.2027/0.0211 | | 0.1603/0.0214 | |  |
| 70 | 0.2 | | | 0.7150/0.0117 | | 0.7259/0.0179 | 0.2025/0.0190 | | 0.1632/0.0241 | |  |
| 70 | 0.4 | | | 0.7126/0.0118 | | 0.7216/0.0209 | 0.2022/0.0166 | | 0.1608/0.0228 | |  |
| 70 | 0.6 | | | 0.7164/0.0202 | | 0.7136/0.0211 | 0.2038/0.0262 | | 0.1610/0.0279 | |  |
| 70 | 0.8 | | | 0.7177/0.0171 | | 0.7130/0.0185 | 0.2119/0.0200 | | 0.1604/0.0218 | |  |
| 70 | 1 | | | 0.7079/0.0111 | | 0.7265/0.0171 | 0.2033/0.0183 | | 0.1629/0.0249 | |  |
| 80 | 0 | | | 0.7151/0.0327 | | 0.6966/0.0349 | 0.2068/0.0297 | | 0.1383/0.0207 | |  |
| 80 | 0.2 | | | 0.7271/0.0129 | | 0.7251/0.0188 | 0.2274/0.0083 | | 0.1574/0.0217 | |  |
| 80 | 0.4 | | | 0.7315/0.0111 | | 0.7118/0.0163 | 0.2266/0.0186 | | 0.1517/0.0142 | |  |
| 80 | 0.6 | | | 0.7399/0.0102 | | 0.7158/0.0178 | 0.2327/0.0138 | | 0.1557/0.0214 | |  |
| 80 | 0.8 | | | 0.7318/0.0124 | | 0.7192/0.0200 | 0.2344/0.0127 | | 0.1614/0.0213 | |  |
| 80 | 1 | | | 0.7384/0.0120 | | 0.7185/0.0181 | 0.2299/0.0167 | | 0.1525/0.0170 | |  |
| 90 | 0 | | | 0.7376/0.0301 | | 0.7070/0.0238 | 0.2318/0.0325 | | 0.1564/0.0146 | |  |
| 90 | 0.2 | | | 0.7398/0.0223 | | 0.7212/0.0194 | 0.2410/0.0268 | | 0.1678/0.0103 | |  |
| 90 | 0.4 | | | 0.7469/0.0212 | | 0.7168/0.0135 | 0.2444/0.0303 | | 0.1646/0.0075 | |  |
| 90 | 0.6 | | | 0.7459/0.0173 | | 0.7139/0.0135 | 0.2393/0.0303 | | 0.1647/0.0148 | |  |
| 90 | 0.8 | | | 0.7529/0.0189 | | 0.7205/0.0191 | 0.2512/0.0294 | | 0.1706/0.0126 | |  |
| 90 | 1 | | | 0.7526/0.0186 | | 0.7166/0.0188 | 0.2482/0.0269 | | 0.1629/0.0106 | |  |

Table S11 Four RBP-RNA complexes in Figure 4b.

| PDB ID | RBP Sequence | RNA Sequence |
| --- | --- | --- |
| 4WQU | PLDVALKRKYYEEVRPELIRRFGYQNVWEVPRLEKVVINQGLGEAKEDARILEKAAQELALITGQKPAVTRAKKSISNFKLRKGMPIGLRVTLRRDRMWIFLEKLLNVALPRIRDFRGLNPNSFDGRGNYNLGLREQLIFPEITYDMVDALRGMDIAVVTTAETDEEARALLELLGFPFRK | UCCCCCGUGCCCAUAGCGGCGUGGAACCACCCGUUCCCAUUCCGAACACGGAAGUGAAACGCGCCAGCGCCGAUGGUACUGGGCGGGCGACCGCCUGGGAGAGUAGGUCGGUGCGGGGGA |
| 4WQU | RLTAYERRKFRVRNRIKRTGRLRLSVFRSLKHIYAQIIDDEKGVTLVSASSLALKLKGNKTEVARQVGRALAEKALALGIKQVAFDRGPYKYHGRVKALAEGAREGGLEF | UCCCCCGUGCCCAUAGCGGCGUGGAACCACCCGUUCCCAUUCCGAACACGGAAGUGAAACGCGCCAGCGCCGAUGGUACUGGGCGGGCGACCGCCUGGGAGAGUAGGUCGGUGCGGGGGA |
| 5OT7 | PRLKVKLVKSPIGYPKDQKAALKALGLRRLQQERVLEDTPAIRGNVEKVAHLVRVEVVE | UCCCCCGUGCCCAUAGCGGCGUGGAACCACCCGUUCCCAUUCCGAACACGGAAGUGAAACGCGCCAGCGCCGAUGGUACUGGGCGGGCGACCGCCUGGGAGAGUAGGUCGGUGCGGGGGG |
| 6UCQ | MEYRLKAYYREGEKPSALRRAGKLPGVMYNRHLNRKVYVDLVEFDKVFRQASIHHVIVLELPDGQSLPTLVRQVNLDKRRRRPEHVDFFVLSDEPVEMYVPLRFVGTPAGVRAGGVLQEIHRDILVKVSPRNIPEFIEVDVSGLEIGDSLHASDLKLPPGVELAVSPEETIAAVVPPEDVEKLAE | UCCCCCGUGCCCAUAGCGGCGUGGAACCACCCGUUCCCAUUCCGAACACGGAAGUGAAACGCGCCAGCGCCGAUGGUACUGGGCGGGCGACCGCCUGGGAGAGUAGGUCGGUGCGGGGGA |
